# A Systematic Review of Complement-Targeting Therapies for Geographic Atrophy: Insights into Advanced Drug Delivery Systems and Gene-Based Approaches

**DOI:** 10.3390/pharmaceutics18101269

**Published:** 2026-10-07

**Authors:** Paula Fernández-Muro, Ana del Pozo-Rodríguez, María Ángeles Solinís, Alicia Rodríguez-Gascón

**Affiliations:** 1 Pharmacokinetic, Nanotechnology and Gene Therapy Group (PharmaNanoGene), Faculty of Pharmacy, Lascaray Research Centre, University of the Basque Country EHU, Paseo de la Universidad n° 7, 01006 Vitoria-Gasteiz, Spain; paula.fernandez@ehu.eus (P.F.-M.); ana.delpozo@ehu.eus (A.d.P.-R.); marian.solinis@ehu.eus (M.Á.S.); 2 Microbiology, Infectious Disease, Antimicrobial Agents, and Gene Therapy, Bioaraba Health Research Institute, C/Jose Atxotegi Kalea s/n, 01009 Vitoria-Gasteiz, Spain

**Keywords:** macular degeneration, geographic atrophy, complement system, avacincaptad pegol, pegcetacoplan, intravitreous injection, nanotechnology, gene therapy

## Abstract

**Background/Objectives:** Geographic atrophy (GA), an advanced form of dry age-related macular degeneration (AMD), causes irreversible central vision loss and has limited treatment options. Complement dysregulation plays a pivotal role in GA pathogenesis. This systematic review synthesizes the efficacy, safety, tolerability, and pharmacokinetics of approved and investigational complement-targeting therapies, alongside emerging drug delivery systems and gene-based approaches. **Methods:** Following PRISMA guidelines, PubMed, Embase, and Cochrane were searched and studies screened against predefined eligibility criteria. Risk of bias and pharmacokinetic quality were assessed using the Newcastle–Ottawa Scale and ClinPK Statement checklist, respectively. **Results:** Thirty-one studies evaluating C3, C5, factor D, and factor B inhibitors; recombinant factor H; soluble CD59 gene therapy; and an indirect anti-amyloid-β strategy were analyzed. Only two agents are FDA-approved: pegcetacoplan (C3 inhibitor) and avacincaptad pegol (C5 inhibitor). They both significantly, albeit modestly, slowed GA lesion growth. However, both increased the risk of exudative AMD conversion and required frequent intravitreal injections. Other complement targets showed limited efficacy or failed to meet clinical endpoints. Pharmacokinetic data, largely derived from serum measurements and model-based predictions, suggest that intravitreal half-life and injection volume dose limitations may complicate the translational development of complement inhibitors. **Conclusions:** Current evidence indicates a modest, agent-, dose-, and regimen-dependent slowing of GA anatomical progression. Clinically meaningful visual function benefit has not been firmly established, although post hoc analyses suggest possible benefits for photoreceptor structure and vision-related risk. Gene therapy and advanced intraocular delivery systems have emerged as potential strategies, but further research must determine whether they can provide sustained, multitarget, patient-centered treatment with acceptable ocular and systemic safety.

## 1. Introduction

Age-related macular degeneration (AMD) is a complex degenerative disease of the macula resulting in progressive vision loss [1]. It is among the most frequent causes of visual impairment in adults [2]. According to a systematic analysis for the Global Burden of Disease (GBD), vision impairment due to AMD affected approximately 8 million individuals in 2021, with projections indicating an increase to 21.34 million by 2050 [3].

The risk of AMD is influenced by a complex interplay of both non-modifiable and modifiable factors. Strong and consistent associations have been reported for advancing age and smoking, as well as for lifestyle and systemic conditions such as diet, diabetes, and hypertension. Several circulating biomarkers, including carotenoids, C-reactive protein (CRP), and high-density lipoprotein cholesterol (HDL-C), have also been linked to AMD, although the strength of these associations varies across studies [4].

The early stage of AMD is characterized by the accumulation of extracellular deposits (drusen) between the retinal pigment epithelium (RPE) and Bruch’s membrane [5]. As the disease advances, it may progress to either the neovascular (“wet”) or non-neovascular (“dry”) form, the latter of which can ultimately lead to geographic atrophy (GA) [6]. Wet AMD involves recruitment of immune cells to the damaged macula and secretion of proinflammatory and proangiogenic cytokines, particularly vascular endothelial growth factor (VEGF), resulting in hemorrhage and fluid leakage, which are associated with progressive degeneration of the RPE and loss of photoreceptors [5,7]. The dry variant, accounting for the majority of AMD cases, is characterized in early and intermediate stages by the accumulation of drusen, degeneration of the retinal pigment epithelium (RPE), and progressive loss of photoreceptors [8]. Dry AMD can evolve to GA, with progressive degeneration of the RPE, the choroid, and the outer retina [6,8]. Patients with dry AMD, particularly in advanced stages such as GA, can experience substantial visual impairment, although the nature and severity of symptoms vary among individuals [9,10]. In a recent study involving patients with GA, the more frequent manifestations reported by the patients (>50%) included poor light adaptation, blurred vision, defective color vision, sensitivity to brighter light, progressive visual loss, restrictive vision fields, and poor contrast vision. Other less frequent but more disturbing symptoms included night blindness, fatigue, line distortion, dry eyes, and sleep disturbance [11].

The treatment of AMD, specifically the advanced forms, is very challenging; in fact, current therapies are targeted toward managing symptoms and slowing the progression of the disease, rather than achieving a cure. For neovascular AMD, anti-VEGF drugs, such as aflibercept, ranibizumab, and bevacizumab among others, administered by repeated intravitreal (IVT) injections, have enabled the management of choroidal neovascularization and macular exudation [12,13,14]. This therapeutic success stands in marked contrast to the dry form of AMD, particularly its advanced stage, GA, for which pharmacological options remain very limited. Antioxidant multivitamin and mineral supplementation are the only available tools that may slow progression from earlier to later stages of the disease, and a healthy diet with a variety of fresh fruit and vegetables may also have many benefits [15]. Despite the progressive and debilitating nature of this condition, achieving effective therapeutic modulation has proved challenging, leaving a substantial unmet medical need for patients worldwide. Several mechanisms are implicated in the development and progression of GA, and a range of approaches have emerged as potential therapeutic targets, including anti-inflammatory and neuroprotective agents, visual cycle modulators, anti-apoptotic drugs, antioxidants, and mitochondrial enhancers [8]. Beyond these approaches, dysregulation of the complement system has emerged as a particularly well-supported driver of disease progression, and therapeutic research has consequently focused increasingly on modulating the complement pathway.

Growing evidence indicates that complement dysregulation plays a central role in the development and progression of GA [16]. The complement system is a key component of innate immunity and can be activated through the classical, lectin, and alternative pathways [17]. Although the classical and lectin pathways were initially characterized as mechanisms for pathogen recognition, accumulating evidence indicates that both pathways can also be triggered by endogenous danger-associated molecular patterns (DAMPs), including apoptotic cells, oxidatively modified molecules, protein aggregates, and extracellular debris [1,18,19,20]. In the aging retina, the progressive accumulation of such stimuli within drusen and the RPE promotes chronic local complement activation in the absence of infection [21,22]. In contrast to the classical and lectin pathways, the alternative pathway is constitutively active at a low level through a process termed tickover of C3 [23,24]. Because of constant tickover, the alternative pathway continuously requires some level of control. This control can be overwhelmed by specific activators of the alternative complement pathway, elevated levels of alternative pathway components, or insufficient function of complement regulatory proteins such as complement Factor H (CFH) [23,24].

**Figure 1:**
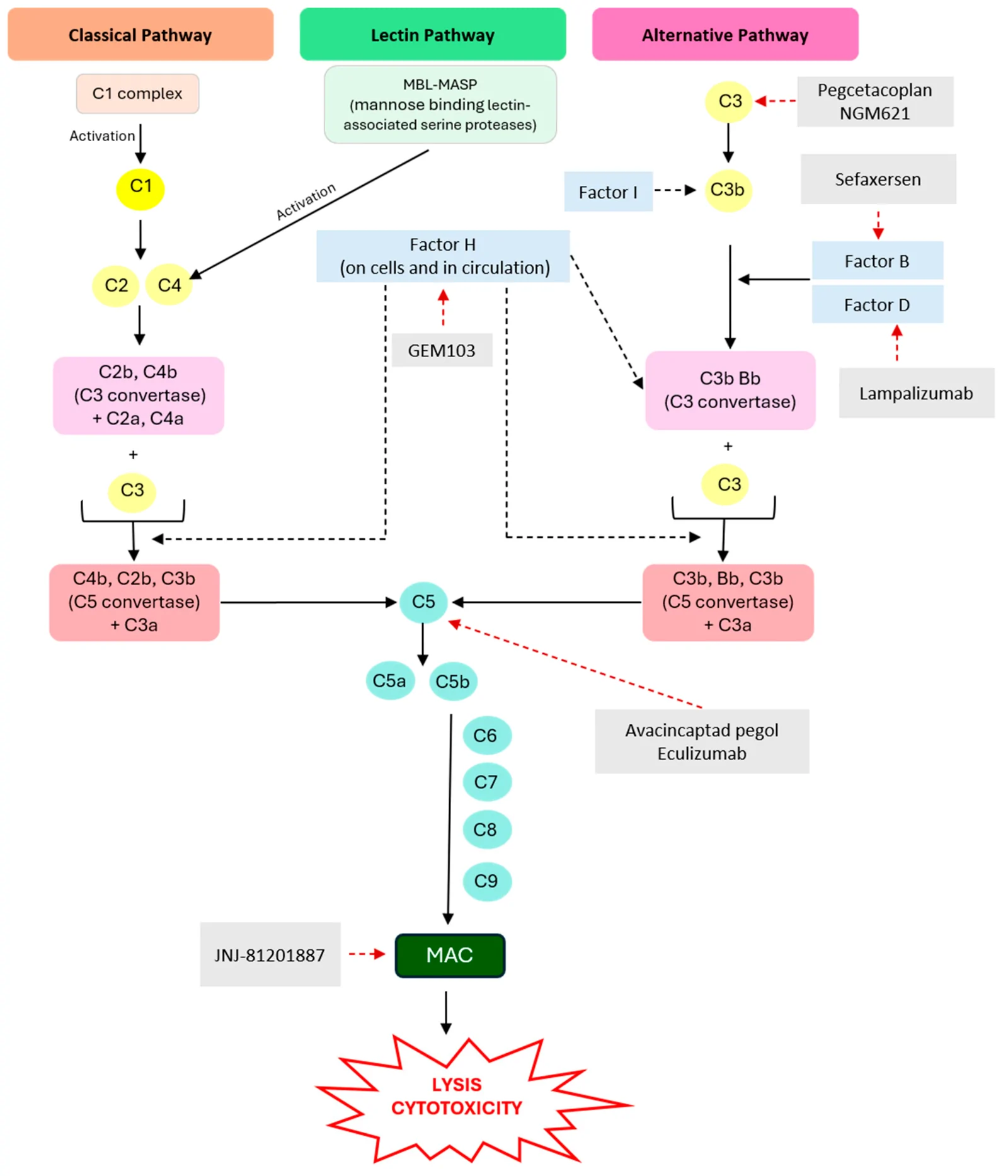
Overview of the complement cascade and drug candidates evaluated in human clinical research, with red arrows pointing to their molecular site of action within the cascade [6,27]. References for clinical research into drug candidates: GEM103 [28], sefaxersen [29], lampalizumab [30], pegcetacoplan [31], eculizumab [32], and avacincaptad pegol [33], NGM621 [34], and JNJ-81201887 [35].

Regardless of the initiating pathway, following activation, a number of proteolytic activation steps lead to the formation of a C3-convertase that cleaves C3 into the pro-inflammatory anaphylatoxin C3a and the opsonin C3b. The anaphylatoxin C3a binds to C3a receptors on immune cells, whereas C3b is deposited on foreign and/or host surfaces. Unless C3b is inactivated by Factor H (generating iC3b), its production leads to the generation of the C5 convertase, which in turn acts on C5 to generate the pro-inflammatory anaphylatoxin C5a and the cleavage product C5b, which recruits C6, C7, C8, and multiple C9 molecules to form C5b-9 or what is defined as the membrane attack complex (MAC) [23,25,26]. Figure 1 features the complement cascade pathway, including therapeutic interventions in clinical evaluation [6,27,28,29,30,31,32,33,34,35].

Complement activation may directly contribute to the formation and growth of drusen [6,22]. In fact, drusen contain numerous complement components, including C3, C5, C9, and the MAC, as well as complement regulatory proteins such as CFH and vitronectin (also known as complement S-protein) [6,36]. Collectively, these mechanisms provide a strong biological rationale for complement modulation as a therapeutic strategy in AMD. In fact, the complement cascade represents the primary target of the only two therapies currently approved for the treatment of geographic atrophy secondary to AMD by the Food and Drug Administration (FDA) of the United States and by the Australian Therapeutic Goods Administration (TGA). Pegcetacoplan (Syfovre), which acts upstream by inhibiting C3 [31], was approved in 2023 by the FDA [37] and in 2025 by the TGA [38]. Avacincaptad pegol, which acts downstream by inhibiting C5 activation [33], was approved in 2023 by the FDA [39] and in 2025 by the TGA [40]. Existing network meta-analyses and systematic reviews have already quantified the comparative efficacy and safety of these two approved agents, showing that both significantly reduce GA lesion growth relative to sham without conferring a demonstrable visual benefit [41,42].

Unfortunately, translating these biological mechanisms into global clinical success has faced significant hurdles. Unlike in the United States, these drugs have not achieved marketing authorization in Europe. The European Medicines Agency (EMA) declined authorization for pegcetacoplan [43], citing a lack of clinically meaningful benefit alongside concerns related to the risks associated with repeated IVT administration [43]. In the case of avacincaptad pegol, in 2024, Astellas Pharma Europe B.V. withdrew its application for a marketing authorization of Izelvay for the treatment of GA caused by AMD [44]. Based on the review of the data and the company’s response to the Agency’s questions, the EMA concluded at the time of withdrawal that a positive balance of the medicine’s benefits and risks could not be established for this medicine. Beyond these two drugs, several other compounds targeting the complement cascade have been evaluated or are currently under investigation in clinical trials. However, the European regulatory landscape and the therapeutic limitations identified to date highlight the urgent need for continued innovation aimed at achieving more effective and safer modulation of complement activity. A comprehensive understanding of the challenges encountered during clinical development is therefore essential to guide the design of next-generation therapeutic strategies.

Addressing these unmet needs will require the development of innovative therapeutic strategies capable of achieving more precise and sustained modulation of the complement system while minimizing treatment burden and adverse effects. Among the approaches currently under investigation, advanced drug delivery platforms, gene-based therapies, and other emerging technologies may offer new opportunities to improve therapeutic outcomes [8,45]. Evaluating the strengths and limitations of these evolving strategies will be essential for guiding the development of safer, more effective, and longer-lasting treatments for GA.

The primary objective of this work was to synthesize, through a systematic review, the available evidence on the efficacy, safety (objective adverse effects), tolerability (the extent to which a drug’s adverse effects can be subjectively tolerated by patients), and pharmacokinetics of drugs recently approved or under clinical development for the treatment of GA through modulation of the complement system. A secondary objective was to provide, through a narrative review, a discussion of emerging therapeutic strategies that may help overcome the current limitations of complement-targeted therapies and improve their clinical performance, including advanced drug delivery systems and gene-based approaches.

## 2. Materials and Methods

### 2.1. Systematic Review

#### 2.1.1. Data Source and Search Strategy

The systematic review was conducted in accordance with the 2020 Preferred Reporting Items for Systematic Reviews and Meta-Analysis (PRISMA) guidelines [46,47]. The protocol was registered on the Open Science Framework (OSF) during identification of studies stage and before screening (https://doi.org/10.17605/OSF.IO/SUQTN, 3 October 2026). The review questions were formulated using the PICOS framework [48] as follows: Which complement-modulating drugs have been clinically evaluated for the treatment of GA, and what evidence is available regarding their efficacy, safety, tolerability, and pharmacokinetics?

A systematic literature search was performed in three electronic databases: PubMed, Embase, and the Cochrane Library. The search strategy combined controlled vocabulary (MeSH in PubMed; Emtree in Embase) with free-text title/abstract terms, using the disease concept ‘Geographic Atrophy’/’Macular Degeneration’ combined with ‘Complement System Proteins’ and related terms. The search was restricted to human clinical studies. To make the search as expansive as possible, the systematic search was conducted up to and including 8 July 2026. The full search strategy for each database is provided in Table S1.

In addition, the reference lists of relevant reviews and original research articles were manually screened to identify further eligible studies. Furthermore, clinical trial registry entries with available data but no associated peer-reviewed publications were also included for analysis. Clinical trial information was retrieved from ClinicalTrials.gov (https://clinicaltrials.gov/) (accessed on 20 September 2026).

#### 2.1.2. Eligibility Criteria

Studies were selected according to the following PICOS criteria.

- Participants were patients diagnosed with GA, primarily secondary to AMD, who received an approved or investigational complement-modulating therapy; healthy-volunteer studies were eligible for safety, tolerability, and pharmacokinetic or pharmacodynamic outcomes and were excluded for the clinical efficacy synthesis.
- Intervention included any therapeutic agent targeting a component of the complement cascade.
- Comparisons were made with sham treatments, placebos, or other control groups, where applicable.
- Outcomes were efficacy, safety, tolerability, and pharmacokinetics.
- Study designs were prospective, interventional, controlled clinical trials.

The inclusion criteria were original articles published in English and conducted in humans, including both healthy volunteers and patients with GA. Eligible studies were required to evaluate approved or investigational therapies targeting the complement cascade and to report data on efficacy, safety, tolerability, or pharmacokinetics. Clinical trial registry records without peer-reviewed publications were used as supplementary evidence.

The exclusion criteria were review articles, preprints, editorials, letters to the editor, expert opinions, in vitro studies, animal studies, cost-effectiveness studies, case reports, conference abstracts, and clinical trials without available results.

#### 2.1.3. Study and Data Extraction

All the authors of the work (P.F.M, A.R.G., M.Á.S., A.d.P.-R.) independently in duplicate screened first the title and abstract and later the full-text of eligible studies after excluding those based on abstract and title. Two authors (P.F.M, A.R.G.) independently completed data extraction in duplicate, and data were then reviewed by the other two authors (M.Á.S., A.d.P.-R.). Disagreements between authors (any mismatch between the extractors or between an extracted value and the source publication, regarding sample size, dosing regimen, administration route, follow-up duration, or reported outcomes) were resolved by joint discussion of all four authors against the original publication until full consensus was reached.

Data on drug name, developer, authors and year of publication, study design, clinical trial identifier, administration route, duration of therapy, number of included subjects, mean age and sex of the subjects, follow-up duration, outcome description, and clinical outcomes were extracted.

#### 2.1.4. Data Synthesis

The primary objective of this systematic review was to synthesize the available clinical evidence on the efficacy, safety, tolerability, and pharmacokinetics of complement-modulating drugs for GA.

Questions related to the therapeutic achievements, current limitations, unexplored complement targets, and possible alternative strategies were later addressed through a narrative critical discussion based on the findings of the included studies.

#### 2.1.5. Quality Assessment and Risk of Bias

The quality assessment of the studies included in the review was carried out using the Newcastle–Ottawa Scale (NOS) [49]. All the authors (P.F.M., A.d.P.-R., M.Á.S. and A.R.G.) independently assessed the articles and compared their results in duplicateand any disagreement was solved by consensus. In the NOS score, the studies were scored across three domains: selection (four questions), comparability (two questions), and outcome (three questions). For the studies including post hoc analyses that were not prespecified in the protocol, no star was awarded in the selection and comparability domains of the Newcastle–Ottawa Scale due to the increased risk of data-driven results. The NOS assessment was used to identify potential methodological limitations and to inform the narrative interpretation of findings. Studies with lower NOS scores were considered with greater caution when evaluating the overall strength and consistency of the evidence. Table S2 includes the specific criteria applied according to the NOS scale.

As no validated risk-of-bias assessment tool is currently available specifically for PK studies, we used the ClinPK checklist [50] to assess the completeness of reporting of the identified manuscripts focusing primarily on pharmacokinetics (PK), rather than as a formal risk-of-bias or methodological quality instrument. The ClinPK checklist (Table S3) provides a structured framework for evaluating the completeness and quality of reporting in clinical pharmacokinetic studies.

### 2.2. Narrative Review

To identify emerging therapeutic strategies that may overcome the limitations of complement-targeted therapies and enhance their clinical efficacy, a comprehensive literature search was performed using the same databases employed in the systematic review, supplemented by searches in relevant company websites. Only articles published in English were included. The narrative review was not necessarily based on the same eligibility criteria as the systematic review search.

## 3. Results

### 3.1. Literature Search

**Figure 2:**
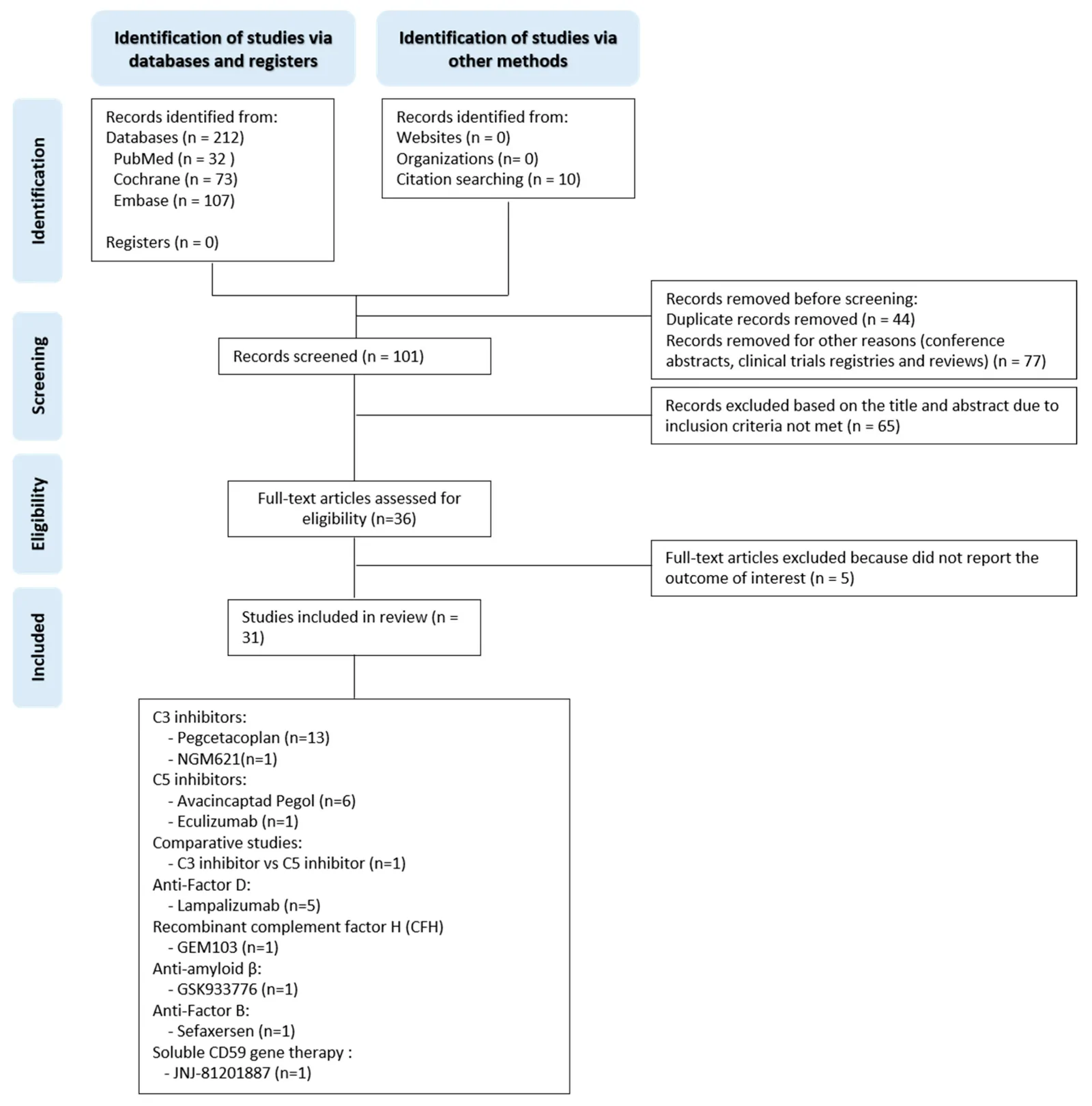
PRISMA flow diagram of the study selection process.

The search strategy applied to the databases to select publications resulted in 212 records comprising 32 in PubMed, 73 in Cochrane, and 107 in Embase. Ten additional records were identified by manually screening the reference lists of relevant studies and were included in the review. Before screening, 44 duplicate records and 77 records removed for other reasons were manually excluded (conference abstracts, clinical trials registries and reviews), leaving 101 records for title and abstract screening. Following this initial screening, 65 records were excluded because they did not meet the predefined inclusion criteria. A total of 36 full-text articles were assessed for eligibility. Of these, 5 publications were excluded because they did not report the outcome of interest. Consequently, 31 articles met the eligibility criteria and were included in the systematic review. Figure 2 presents a flow diagram of the literature search results.

In addition to the 31 articles selected following PRISMA guidelines, we identified 31 clinical trial registry entries with available data but with no associated peer-reviewed publications (Table S5).

### 3.2. Study Characteristics

Most studies involved complement inhibitors such as C3 inhibitors (14 manuscripts involving 2 drugs and 7 clinical trials), C5 inhibitors (7 manuscripts involving 2 drugs and 2 clinical trials), CFD inhibitors (5 manuscripts involving 1 drug and 4 clinical trials), and complement factor B (CFB) inhibitors (1 manuscript involving 1 drug and 2 clinical trials). Other strategies include reducing complement activation by decreasing complement fixation and Fc receptor interaction using an anti-amyloid-β monoclonal antibody (one manuscript involving one drug and one clinical trial), overexpressing CFH (one manuscript involving one drug and one clinical trial) and expressing soluble CD59 (one manuscript involving one product based on gene therapy and one clinical trial). Additionally, one manuscript compared more than one drug (C3 and C5 inhibitors).

#### 3.2.1. Recombinant Complement Factor H

GEM103, a recombinant CFH (rCFH), was evaluated in an open-label, Phase 1 single-ascending-dose trial involving 12 patients with confirmed foveal GA who received a single IVT injection of 50, 100, 250, or 500 µg/eye [28]. No dose-limiting toxicities were reported, even in the highest dose group. CFH levels in aqueous humour increased in a dose-dependent manner, reaching supraphysiological levels at week 1 and remaining above baseline for over 8 weeks in all participants receiving 100 µg/eye or higher. Most subjects showed a reduction in complement activation biomarkers; however, the trial was underpowered to evaluate clinical outcomes due to the study’s sample size and design (Table 1). Although a subsequent study evaluating repeat IVT injections of GEM103 in dry AMD (NCT04643886) was conducted, it was terminated due to a lack of additional benefit, according to the sponsor-reported reason (Table S5).

#### 3.2.2. Complement Factor B Inhibitors

Sefaxersen (IONIS-FB-LRx) is a GalNAc-conjugated antisense oligonucleotide targeting complement factor B mRNA. The goal of this drug is to maintain lower alternative pathway (AP) activity by reducing systemic factor B levels. In two Phase 1 clinical trials involving healthy volunteers [29] (Table 2), subcutaneous administration achieved a reduction of 38% and 69% in factor B levels after single and repeated doses, respectively. Notably, factor B levels remained suppressed for up to 90 days, supporting a monthly dosing regimen. The reduction in factor B levels correlated with alternative pathway activity but not with classical pathway activity. Since both clinical studies involved healthy volunteers, clinical efficacy was not demonstrated. The Phase 1 results supported the Phase II GOLDEN study (NCT03815825) which evaluated the safety and efficacy of subcutaneous IONIS-FB-LRx administered every 4 weeks in participants with GA secondary to AMD (Table S5). Although the study was completed in 2024, no peer-reviewed results are currently available. Iptacopan, an oral small-molecule CFB inhibitor, is currently being evaluated in an active Phase 2 trial to assess its effect to prevent conversion of early or intermediate AMD eyes to new incomplete RPE and outer retinal atrophy (iRORA) or late AMD (NCT05230537) (Table S5).

#### 3.2.3. Complement Factor D Inhibitors

The main characteristics and key outcomes of published clinical trials evaluating CFD inhibitors are summarized in Table 3. Lampalizumab (formerly FCFD4514S) is an antigen-binding fragment of a humanized monoclonal antibody directed against complement factor D. In a Phase 1a single-dose escalation trial involving 18 patients with GA, IVT administration of lampalizumab (0.1 to 10 mg) was well tolerated, with no dose-limiting toxicity or immunogenicity. Higher doses were not assayed due to formulation limitations of preparing them within a 0.1 mL dose volume [51]. Serum drug concentrations for all dosing cohorts following IVT administration were significantly below levels predicted to transiently impact systemic complement activity. Moreover, vitreous concentrations of anti-CFD in humans could be estimated from the serum elimination half-life (6 days) in a Phase Ia study and preclinical data from cynomolgus monkey studies and were predicted to remain above the level necessary to inhibit CFD activity for 60 days after a 10 mg IVT dose [51]. In the Phase 2 MAHALO study (n = 123), 10 mg IVT lampalizumab reduced GA lesion area progression by 20% monthly compared to sham at 18 months, an effect that reached 44% in carriers of the complement factor I (CFI) risk allele (a targeted, exploratory genetic analysis was included to identify CFI carriers and to evaluate if the complement pathway may affect GA progression and the treatment response to lampalizumab) [52]. The serum concentrations of lampalizumab remained too low to temporarily suppress the alternative complement pathway (based on preclinical data), and no systemic accumulation was found. By measuring the drug and CFD levels in the aqueous humor, the authors predicted concentration and target suppression in the vitreous humor; this simulation revealed that lampalizumab would successfully suppress most active CFD targets for up to 60 days after injection. In another work [53], a population pharmacokinetic/pharmacodynamic (PK/PD) model based on Phase 1 and 2 data predicted that IVT administration of 10 mg every 4 or 6 weeks would achieve near-complete target suppression in the vitreous humor with minimal systemic inhibition.

Despite these promising findings, two identically designed Phase 3 trials CHROMA (n = 906) and SPECTRI (n = 975), failed to demonstrate significant efficacy. Both studies were suspended at the sponsor’s recommendation because the lack of efficacy did not warrant continued IVT injection. Lampalizumab did not slow GA progression or improve visual function overall, and contrary to the MAHALO study, no benefit was observed in CFI-positive subgroups, leading to early study discontinuation [30]. Analyses of complement activity in aqueous humour samples from these trials revealed no strong correlation between lampalizumab concentrations and CFD levels or Bb:CFD ratio over time; moreover, no change in C4 and C3 processing was detected [54]. Additional Phase 2 and 3 extension trials for lampalizumab were completed or terminated without formal peer-reviewed publication (Table S5). 

Danicopan (ALXN2040), an oral small-molecule inhibitor of CFD, is currently being evaluated in an active Phase 2 trial (NCT05019521) assessing doses up to 400 mg daily over 52 weeks (Table S5).

#### 3.2.4. C5 Inhibitors

The main characteristics and key outcomes of published clinical trials evaluating C5 inhibitors are summarized in Table 4. Two C5 inhibitors with published clinical trial results have been evaluated (eculizumab and avacincaptad pegol). Additionally, tesidolumab (LFG316), an anti-C5 monoclonal antibody administered intravitreally, was evaluated in Phase 1 (NCT01255462) and a Phase 2 (NCT01527500) trials without formal peer-reviewed publications (Table S5).

Eculizumab is a humanized monoclonal antibody derived from the murine anti-human C5 antibody. In an exploratory study (COMPLETE) [32] designed as a pilot clinical trial whose main outcome was the change in area of GA at week 26, eculizumab administered as intravenous infusion, although well tolerated, did not significantly decrease the growth rate of GA. Measurable serum levels of eculizumab were confirmed between onset of treatment and week 26. While in patients receiving placebo injections, circulating levels of eculizumab were undetectable and C5 activity was normal, in patients receiving eculizumab, C5 activity decreased to less than 9% of normal levels by week 1 and to less than 1% by week 2. The authors attributed the lack of efficacy to several factors: (i) the complement system may play a greater role in earlier stages rather than in GA progression, (ii) the dose of eculizumab was too low, or (iii) the drug should have been administered by IVT route to achieve sufficient concentrations in the retina or RPE.

Avacincaptad pegol is a pegylated RNA aptamer with a potent and specific capacity to inhibit cleavage of C5 into C5a and C5b, preventing the formation of key terminal fragments, regardless of the initial activation pathway (alternative, classical, or lectin). Through this mechanism, it aims to attenuate complement-mediated retinal cell degeneration and slow the progression of GA associated with AMD. Avacincaptad pegol has been assayed in two main clinical trials: GATHER 1 and GATHER 2. In the GATHER 1 study, a randomized pivotal Phase 2/3 trial, patients received avacincaptad pegol at doses of 1 mg, 2 mg or 4 mg, administered monthly by IVT route for 12 months. Due to the high viscosity, the dose of 4 mg was administered as two separate IVT injections of 2 mg in 100 µL volume. At the dose level of 2 and 4 mg, avacincaptad pegol led to a reduction in the mean GA growth rate over 12 months of 27.4% and 27.8% compared with the corresponding sham controls, respectively [55]. At month 18, avacincaptad pegol showed 28.1% and 30.0% mean reduction in lesion growth for the 2 mg and 4 mg groups, respectively [56]. However, no benefit on the mean change in BCVA (best-corrected visual acuity) or low luminance BCVA (LL-BCVA) was observed. Avacincaptad pegol was well tolerated over 18 months, although the incidence of total and injection-related adverse events was higher with the 4 mg dose, likely owing to the increased injection volume (two 2 mg injections, 200 µL total volume vs. one 2 mg injection, 100 µL), rather than the dose. Taking into account that the 2 mg dose presented similar efficacy and a better safety profile than 4 mg in the GATHER 1 study and that two IVT injections are necessary to administer the dose of 4 mg, in a confirmatory Phase 3 pivotal trial (GATHER 2), only the 2 mg dose was administered. Avacincaptad pegol 2 mg administered monthly was well tolerated and demonstrated significantly slower GA growth over 12 months, but no effect on BCVA and LL-BCVA was detected [57]. The change in GA area growth was observed in all patient demographic and baseline characteristics, including both male and female subgroups. Injecting the same dose level either every month or every other month continued to decrease GA growth versus sham group over 2 years without safety signal compared with year 1 [33]. In both the GATHER 1 and GATHER 2 study, the incidence of macular neovascularization was higher with avacincaptad pegol 2 mg than with sham. An active Phase 3 trial (NCT05536297) is currently evaluating monthly administration of 2 mg avacincaptad pegol up to month 17 (Table S5).

In a later 12-month post hoc study of the GATHER 1 and GATHER 2 trials [58], fewer study eyes treated with avacincaptad pegol 2 mg lost ≥10, ≥15, or ≥20 BCVA letters from baseline over 12 months than eyes in the sham group (11.6%, 4.0%, and 1.6% vs. 14.1%, 7.6%, and 4.5%, respectively). The risk of persistent ≥ 15 BCVA ETDRS letter loss through month 12 was also lower with avacincaptad pegol 2 mg than with sham (3.4% vs. 7.8%). In addition, fewer avacincaptad pegol-treated eyes reached the visual acuity threshold for driving ineligibility by month 12. To better understand the impact of the treatment with avacincaptad pegol on the progression from iRORA to complete RPE and outer retinal atrophy (cRORA) and from drusen to iRORA and/or cRORA in eyes with GA, a recent post hoc analysis of the GATHER 1 trial was conducted [59]. Results revealed that in patients receiving the avacincaptad pegol, a lower proportion of eyes progressed from drusen to iRORA/cRORA and from iRORA to cRORA, in comparison to sham. Moreover, the study supports the sustained reduction in GA lesion growth after 18-month treatment with 2 mg of avacincaptad pegol. Because these analyses were not prespecified in the trial protocols, their findings should be regarded as supportive and hypothesis-generating rather than confirmatory.

#### 3.2.5. C3 Inhibitors

The main characteristics and key outcomes of published clinical trials evaluating C3 inhibitors are summarized in Table 5. Two C3 inhibitors with published clinical trial results have been evaluated for GA: NGM621 and pegcetacoplan.

NGM621 is a humanized immunoglobulin G1 monoclonal antibody with a two-point mutation in the Fc region to eliminate effector function that potently binds C3 and prevents its cleavage into C3a and C3b. In a first-in-human Phase I trial (NCT04014777), IVT administration of NGM621 was well-tolerated after single ascending doses (2 mg, 7.5 mg, or 15 mg) and multiple doses (two 15-mg doses 4 weeks apart), with no drug-related serious adverse events or choroidal neovascularization [34]. The 15 mg dose was selected as the maximal deliverable dose in a 100 µL volume. Pharmacokinetic analyses indicated that NGM621 serum exposures appeared to be dose-proportional, and no antidrug antibodies were identified. Moreover, based on extrapolation from studies in the cynomolgus monkey, systemic NGM621 exposures did not reach the threshold necessary to inhibit the complement system. Visual acuity and GA lesion area remained stable throughout the 12-week follow-up. However, the limited sample size, short follow-up, and absence of a control group preclude conclusions regarding efficacy. Subsequently, the Phase II CATALINA trial (NCT04465955) evaluated NGM621 in a larger cohort; nevertheless, registry-reported results indicated no significant reduction in GA progression compared with sham (Table S5).

Pegcetacoplan is a pegylated peptide that binds to both C3 and C3b, preventing the formation of C3 convertase and thereby inhibiting complement activation. The Phase II FILLY trial (NCT02503332) [60] was the first randomized study to evaluate the efficacy and safety of IVT pegcetacoplan in patients with GA secondary to AMD. Monthly and every-other-month (EOM) administration of 15 mg pegcetacoplan over 12 months reduced GA lesion growth by 29% and 20%, respectively, compared with sham, with a more pronounced effect between months 6 and 12. Lesion growth accelerated following treatment discontinuation, indicating that sustained complement inhibition is required to maintain efficacy. No significant differences in visual acuity decline were observed. Safety analyses revealed a dose-dependent increase in the incidence of exudative AMD (eAMD) in pegcetacoplan-treated eyes. A pharmacogenetic analysis showed that AMD-associated variants in CFI, CFH, C2/CFB, and C3 did not modify the therapeutic effect of pegcetacoplan. A subsequent post hoc analysis [61] identified baseline eAMD in the fellow eye and non-exudative macular neovascularization in the study eye as key predictors for eAMD development. Furthermore, extrafoveal GA lesion location, BCVA ≥ 20/60, and low-luminance deficit were identified as risk factors associated with greater GA lesion growth [62]. Multiple post hoc imaging studies based on FILLY data [63] provided further structural insights. OCT-based evaluations confirmed the reduction in RPE atrophy progression and demonstrated preserved ellipsoid zone (EZ) and external limiting membrane (ELM) integrity.

Deep learning-assisted spectral-domain OCT analysis [64] demonstrated significantly less photoreceptor loss in patients receiving monthly or EOM pegcetacoplan, together with reduced photoreceptor thinning in the monthly treatment group, suggesting that complement inhibition may preserve photoreceptor integrity beyond its effects on RPE atrophy. Similarly, Pfau et al. [65] reported reduced thinning of the outer retina in a dose-dependent manner, with monthly treatment preserving the outer nuclear layer, photoreceptor inner segments, and outer segments outside the RPE atrophy junctional zone. In addition, Nittala et al. [66] found that pegcetacoplan reduced the progression from iRORA to cRORA, suggesting a potential benefit during earlier stages of AMD before the development of established GA.

The pivotal Phase 3 trials OAKS (NCT03525613) and DERBY (NCT03525600) confirmed these findings in over 1200 patients receiving 15 mg pegcetacoplan monthly or EOM for 24 months [67]. In OAKS, monthly and EOM administration reduced GA lesion growth by 21% and 16%, respectively, at month 12, with corresponding reductions of 22% and 18% at month 24. In DERBY, statistically significant treatment effects emerged after longer follow-up, with reductions of 19% and 16% in GA growth at month 24 for monthly and EOM dosing, respectively. In both trials, treatment effects were greater in eyes with non-subfoveal lesions than in those with subfoveal involvement. Despite the anatomical benefits, no significant differences in visual function endpoints were observed after 24 months. The incidence of eAMD remained higher in pegcetacoplan-treated groups, consistent with previous findings from the Phase II FILLY trial. A post hoc deep learning-based OCT analysis of pooled OAKS and DERBY data further demonstrated that pegcetacoplan reduced photoreceptor degeneration to a greater extent than RPE loss [68]. Moreover, the baseline difference between the areas of ellipsoid zone (EZ) loss and RPE atrophy, reflecting photoreceptor degeneration beyond the atrophic lesion, was identified as a potential biomarker of disease activity and treatment response [68].

As a continuation of the parental Phase III trials (OAKS and DERBY), long-term outcomes and safety were evaluated in the GALE (NCT04770545) and APL2-103 (NCT03777332) extension studies [69,70]. These studies followed patients for up to a total of 36 and 24 months, respectively. Continued treatment maintained the reduction in GA lesion growth, with greater efficacy in non-subfoveal GA, while the safety profile remained consistent with OAKS and DERBY, showing no new safety signals and a stable incidence of exudative AMD [69,70]. In exploratory analyses, monthly pegcetacoplan treatment was associated with significantly better preservation of retinal function, as assessed by microperimetry at months 30 and 36 of total follow-up in the GALE study, suggesting that the preserved retinal tissue may retain functional activity [69].

Pharmacokinetic and PK/PD modelling studies further characterized IVT pegcetacoplan. A population pharmacokinetic model for pegcetacoplan after IVT injection was developed using serum concentration data from the FILLY and DERBY trials together with two Phase II studies in neovascular AMD (NCT02461771 and NCT03465709) [71]. From serum concentration–time data, the ocular disposition of the drug after IVT administration was inferred. The model showed that systemic exposure of pegcetacoplan following IVT administration is absorption-limited, with a vitreous-to-serum absorption half-life of 13.1 days, substantially longer than the 4.51-day serum elimination half-life. Vitreous exposure was predicted to be more than 1300-fold higher than serum exposure, with steady-state serum exposure remaining below the level required for systemic pharmacodynamic effects. In a separate study, a PK/PD analysis of GA lesion area was conducted using pooled data from 1501 patients from the FILLY, OAKS, and DERBY trials [31]. A dose–response model first estimated 0.80-fold and 0.83-fold reductions in GA lesion growth rate with pegcetacoplan monthly and EOM, respectively, versus sham. Additionally, a relationship between vitreous humor concentration and GA lesion growth rate was quantified as 2.6% per unit of log-transformed vitreous pegcetacoplan concentration. The PK/PD model was subsequently used as the basis for analyses of pooled patient-level data from the FILLY, OAKS, and DERBY trials, linking predicted vitreous pegcetacoplan concentrations with reductions in GA lesion growth and supporting the monthly and every-other-month dosing regimens.

Finally, AL-78898A, another IVT C3 inhibitor candidate, was evaluated in a Phase 2 trial (NCT01603043) that was terminated early with no published peer-reviewed results (Table S5).

**Table 5:** Characteristics of the studies involving C3 inhibitors.

Drug (API), Developer	Author, Year, Ref. No.	Clinical Trial ID, Phase, Design	Administration Route, Dose, Duration	Subjects (*n*), Follow Up	Outcome Description	Mean Clinical Outcomes
NGM621 (monoclonal antibody targeting complement C3) NGM Biopharmaceuticals, Inc., South San Francisco, CA, USA.	Wykoff CC, et al. 2022 [34]	NCT04014777 Multicenter, open-label, single ascending dose) and multiple-dose) Phase 1 study.	IVT Single dose: 2 mg, 7.5 mg, and 15 mg Two doses of 15 mg 4 weeks apart	15 patients Mean age: 78.6 years Female: 40% Follow up: 12 weeks.	Adverse events, BCVA, LLBCVA, vital signs, clinical laboratory evaluations, GA lesion area, spectral domain OCT, PK/PD and, immunogenicity.	Safe and well tolerated. Mean visual acuity and GA lesion area appeared stable through week 12 for all cohorts.
Pegcetacoplan (PEGylated peptide targeting complement C3) Apellis Pharmaceuticals, Inc., Waltham, MA, USA	Liao DS, et al. 2020 [60]	NCT02503332 (FILLY) Prospective, multicenter, randomized, single-masked, sham-controlled Phase 2 study.	IVT 15 mg monthly or EOM for 12 months	246 patients Mean age: 80 years Female: 62.6% Follow up: 6 months.	Primary outcome: mean change in square root GA lesion area from baseline to month 12, mean change from baseline in GA lesion area without the square root transformation, distance of GA lesion from the fovea, BCVA, LLBCVA, and LL visual acuity deficit (safety and adverse events also considered).	Significant reductions in the growth of GA (29% monthly, 20% EOM) compared with sham treatment. Well tolerated.
Pegcetacoplan (PEGylated peptide-targeting complement C3) Apellis Pharmaceuticals, Inc., Waltham, MA, USA	Wykoff CC, et al. 2021 [61]	Post hoc NCT02503332 (FILLY) Prospective, multicenter, randomized, single-masked, sham-controlled Phase 2 study.	IVT 15 mg monthly or EOM for 12 months	246 patients Mean age: 80 years Female: 62.6% Follow up: 12 weeks.	Time of new eAMD onset in the study eye, history of eAMD in the fellow eye, presence of double-layer sign (DLS) on structural OCT in the study eye, changes in retinal anatomic features by structural OCT and fluorescein angiography (FA), and changes in visual acuity.	Slowed rate of GA growth, associated with an unexpected increased incidence of new-onset eAMD (dose-dependent).
Pegcetacoplan (PEGylated peptide-targeting complement C3) Apellis Pharmaceuticals, Inc., Waltham, MA, USA	Steinle NC, et al. 2021 [62]	Post hoc NCT02503332 (FILLY) Prospective, multicenter, randomized, single-masked, sham-controlled Phase 2 study.	IVT 15 mg monthly or EOM for 12 months	176 patients Age: 78.7 years Female: 62.5% No separate follow-up period reported.	Effects of age, gender, lesion size, focality, location (extrafoveal vs. foveal), pseudodrusen status, BCVA, and LLD) on GA progression at month 12.	Reduced geographic atrophy progression after controlling for key risk factors, including extrafoveal lesions and greater low-luminance deficit.
Pegcetacoplan (PEGylated peptide-targeting complement C3) Apellis Pharmaceuticals, Inc., Waltham, MA, USA	Nittala, MG et al. 2022. [66]	Post hoc NCT02503332 (FILLY) Prospective, multicenter, randomized, single-masked, sham-controlled Phase 2 study.	IVT 15 mg monthly or EOM for 12 months	167 patients Age: 78.7 years Female: 63.5% No separate follow-up period reported.	Proportion of study eyes with at least 1 iRORA at baseline that progressed to cRORA at 6 and 12 months and the proportion of individual baseline iRORA lesions that progressed to cRORA lesions at 6 and 12 months.	Reduced progression from iRORA to cRORA at 12 months compared with sham (RR 0.61 monthly, 0.74 EOM).
Pegcetacoplan (PEGylated peptide-targeting complement C3) Apellis Pharmaceuticals, Inc., Waltham, MA, USA	Pfau M, et al. 2022 [65]	Post hoc NCT02503332 (FILLY) Prospective, multicenter, randomized, single-masked, sham-controlled Phase 2 study.	IVT 15 mg monthly or EOM for 12 months	192 patients Mean age: 79.4 years Female: 63.5% No separate follow-up period reported.	Primary outcome: change from baseline in (standardized) outer nuclear layer (ONL) thickness at the 5.16–contour line at month 12. Secondary outcome: effect of pegcetacoplan on RPE-atrophy progression rate.	Treatment with pegcetacoplan was associated with less outer retinal thinning over time. Monthly dosing preserved more tissue at the ONL, IS, and OS layers beyond the GA junctional zone at month 12.
Pegcetacoplan (PEGylated peptide-targeting complement C3) Apellis Pharmaceuticals, Inc., Waltham, MA, USA	Mai J, et al. 2022 [63]	Post hoc NCT02503332 (FILLY) Prospective, multicenter, randomized, single-masked, sham-controlled Phase 2 study.	IVT 15 mg monthly or EOM for 12 months	113 patients Mean age: 78.7 years Female: 63.5% No separate follow-up period reported.	Correlation of GA areas measured on FAF and OCT. Difference in square root transformed growth rates of RPE, EZ, and ELM loss between treatment groups. Comparison of RPE, EZ, and ELM loss growth between treatment groups on OCT.	Pegcetacoplan slowed RPE atrophy progression and reduced EZ impairment, as detected by OCT, which proved more sensitive than FAF.
Pegcetacoplan (PEGylated peptide-targeting complement C3) Apellis Pharmaceuticals, Inc., Waltham, MA, USA	Riedl S, et al. 2022 [64]	Post hoc NCT02503332 (FILLY) Prospective, multicenter, randomized, single-masked, sham-controlled Phase 2 study.	IVT 15 mg monthly or EOM for 12 months	161 patients Mean age: 78.7 years Female: 63.5% No separate follow-up period reported.	Square-root transformed PR loss area in mm or mm, PR thickness in mm, and PR loss/RPE loss ratio.	Pegcetacoplan significantly reduced PR thinning within the GA junctional zone and surrounding areas, with greater inhibition of photoreceptor loss than RPE loss.
Pegcetacoplan (PEGylated peptide-targeting complement C3) Apellis Pharmaceuticals, Inc., Waltham, MA, USA	Heier JS, et al. 2023 [67]	NCT03525613 (OAKS) NCT03525600 (DERBY) Multicenter, randomized, double-masked, sham-controlled, Phase 3 trials.	IVT 15 mg monthly or EOM for 24 months	OAKS: 614 patients DERBY: 597 patients Mean age: OAKS: 78.5 years DERBY: 78.8 years Female: OAKS: 61% DERBY: 60.6% No separate follow-up period reported.	Primary outcome: change in GA lesion area at 12 months. Secondary outcomes (24 months): reading performance (maximum reading speed, reading independence), visual function (BCVA, mean retinal sensitivity), considering safety.	Pegcetacoplan slowed GA lesion growth in the OAKS and DERBY trials with monthly and EOM dosing, though no differences in visual function were observed at 24 months.
Pegcetacoplan (PEGylated peptide-targeting complement C3) Apellis Pharmaceuticals, Inc., Waltham, MA, USA	Schmidt-Erfurth U, et al. 2025 [68]	Post hoc NCT03525613 (OAKS) NCT03525600 (DERBY) Multicenter, randomized, double-masked, sham-controlled, Phase 3 trials.	IVT 15 mg/0.1 mL monthly, pegcetacoplan 15 mg/0.1 mL EOM, sham monthly, or sham EOM for 24 months	897 patients Mean age: 78 years Female: 60.6% No separate follow-up period reported.	Change in the mean area of RPE loss and EZ loss over time in the pooled sham arms and the pegcetacoplan monthly/pegcetacoplan EOM treatment arms.	Well tolerated. Pegcetacoplan significantly reduced RPE loss (20–27%) and EZ loss (46–53%) versus sham over 24 months. Greater baseline photoreceptor degeneration was associated with faster GA progression and a greater treatment response.
Pegcetacoplan (PEGylated peptide-targeting complement C3) Apellis Pharmaceuticals, Inc., Waltham, MA, USA	Crass, RL et al. 2024 [71]	NCT03525600 (DERBY): multicenter, randomized, double-masked, sham-controlled Phase 3 study. NCT02503332 (FILLY): prospective, multicenter, randomized, single-masked, sham-controlled Phase 2 study. NCT02461771: open-label, non-randomized Phase 1 study. NCT03465709: multicenter, open-label Phase 1b/2 study.	IVT 15 mg monthly, 15 mg EOM, sham monthly, or sham EOM for 24 months	261 patients Mean age: 79.6 years Female 56.7% No separate follow-up period reported	Serum concentrations, PK modeling.	Population PK model adequately described the serum concentration-time profile of pegcetacolplan after IVT administration. First-order absorption from the vitreous to serum, and first order elimination from the serum.
Pegcetacoplan (PEGylated peptide-targeting complement C3) Apellis Pharmaceuticals, Inc., Waltham, MA, USA	Goldberg RA. et al. 2025 [69]	NCT03525600 (DERBY): multicenter, randomized, double-masked, sham-controlled Phase 3 study. NCT02503332 (FILLY): prospective, multicenter, randomized, single-masked, sham-controlled Phase 2 study. NCT02461771: open-label, non-randomized Phase 1 study. NCT03465709: multicenter, open-label Phase 1b/2 study.	IVT 15 mg monthly or EOM for 30 months. Patients of OAKS or DERBY continued the same regimen in GALE. Total time of treatment: 30 months.	790 patients Mean age: 77.4 years Female 59.5% No separate follow-up period reported	Incidence and severity of adverse events. Change from baseline in total GA area and mean rate of change in GA area.	Pegcetacoplan slowed GA lesion growth compared with sham with both monthly and EOM dosing. No significant differences were observed in key visual function endpoints at 24 months.
Pegcetacoplan (PEGylated peptide-targeting complement C3) Apellis Pharmaceuticals, Inc., Waltham, MA, USA	Wykoff CC, et al. 2025 [70]	NCT03525600 (DERBY): multicenter, randomized, double-masked, sham-controlled Phase 3 study. NCT02503332 (FILLY): prospective, multicenter, randomized, single-masked, sham-controlled Phase 2 study. NCT02461771: open-label, non-randomized Phase 1 study. NCT03465709: multicenter, open-label Phase 1b/2 study.	IVT 15 mg monthly or EOM for 36 months	790 patients Mean age: 79.5 years Female: 60% No separate follow-up period reported	Safety and efficacy of long-term pegcetacoplan.	Pegcetacoplan demonstrated sustained slowing of GA lesion growth over 36 months with monthly and EOM dosing. No meaningful improvement in visual function was observed over long-term follow-up.
Pegcetacoplan (PEGylated peptide-targeting complement C3) Apellis Pharmaceuticals, Inc., Waltham, MA, USA	Crass RL et al. 2025 [31]	NCT02503332 (FILLY): multicenter, randomized, single-masked, sham-controlled, Phase 2 trial. NCT03525613 (OAKS) multicenter, randomized, double-masked, sham-controlled, Phase 3 trial. NCT03525600 (DERBY): multicenter, randomized, double-masked, sham-controlled, Phase 3 trial.	IVT Single IVT injections of 4, 10, and 20 mg and multiple IVT injections of 15 mg monthly or EOM.	1501 patients Mean age: 79 years Female: 62% No separate follow-up period reported.	Quantify the progression of lesion area and assess the impact of baseline disease characteristics and pegcetacoplan treatment.	Unilateral, unifocal, and subfoveal GA and >20 intermediate or large drusen groups (≥63 µm) at baseline associated with slower lesion growth. 0.80-fold and 0.83-fold reduction in GA lesion growth with pegcetacolplan monthly or EOM, respectively, versus sham.

GA: geographic atrophy; RPE: retinal pigment epithelium; PR: photoreceptor; EZ: ellipsoid zone; ELM: external limiting membrane; LL: low-luminance; LLD: low-luminance deficit, PEG: polyethylene glicol, IVT: intravitreal, PK/PD: pharmacokinetic/pharmacodynamic, FAF: fundus autofluorescence, BCVA: best corrected visual acuity, LLBCVA: low-luminance best corrected visual acuity, OCT: optical coherence tomography, eAMD: exudative AMD, iRORA: incomplete retinal pigment epithelial and outer retinal atrophy, cRORA: complete retinal pigment epithelial and outer retinal, EOM: every other month, PK: pharmacokinetic.

#### 3.2.6. C3 Inhibitor vs. C5 Inhibitor

To date, only one study has directly compared the real-world outcomes of the two FDA-approved therapies for GA, pegcetacoplan (a C3 inhibitor) and avacincaptad pegol (a C5 inhibitor) (Table 6). This retrospective comparative analysis evaluated patients treated in clinical practice over a 12-month period [72]. After 12 months, both drugs demonstrated comparable clinical outcomes, with no significant differences in GA lesion growth, visual acuity changes, or exudation rates. However, patients treated with avacincaptad pegol received more administrations than those treated with pegcetacoplan (9.05 vs. 5.96), reflecting differences in their FDA-approved dosing schedules (every 4 weeks vs. every 29–60 days, respectively).

#### 3.2.7. Soluble CD59 Gene Therapy

JNJ-81201887 is a recombinant adeno-associated virus serotype 2 (AAV2) vector designed to drive the continuous expression of soluble CD59 (sCD59). CD59 is an endogenous regulator of the complement system that inhibits the incorporation of C9 into the C5b-8 complex, thereby preventing the formation of the membrane attack complex (MAC, C5b-9), a mechanism established in in vitro biochemical and cell-based functional studies of CD59 activity [73]. In this construct, the CD59’s native glycosylphosphatidylinositol membrane anchor is removed, and expression is regulated by a synthetic chicken β-actin promoter. In a Phase 1, open-label, single-center, first-in-human clinical study (NCT03144999), the safety and tolerability of a single IVT injection of JNJ-81201887 (three dose levels) in patients with GA secondary to advanced AMD was evaluated [35] (Table 7). After 24 months of follow-up, all three tested doses showed a safe and tolerable profile. The results aligned with the targeted disease and treatment, with no systemic side effects linked to the therapy. Since the primary objective of this open-label study was the evaluation of safety and tolerability, no definitive conclusions could be drawn about the potential efficacy of this treatment. Currently, JNJ-81201887 is being evaluated in a global Phase 2b trial in patients with GA (NCT05811351). Upcoming efficacy and safety outcomes from this study will be key to establishing its therapeutic profile.

#### 3.2.8. Indirect Complement-Modulating Strategies: Anti-Amyloid β Monoclonal Antibody

In a multicenter Phase 2 clinical trial, the efficacy of GSK933776, a humanized monoclonal antibody directed against the N-terminal amino acids of amyloid β, in slowing the enlargement of GA in patients with GA secondary to AMD was investigated [74] (Table 8). Amyloid β accumulates within drusen and has been shown to impair the regulatory activity of CFI; this loss of regulatory control is proposed to permit sustained, low-grade activation of the alternative pathway [75]. On this basis, GSK933776 was designed as an indirect, mechanistically related strategy that removes amyloid β to restore CFI regulatory function, rather than as a direct inhibitor of a complement cascade component such as C3, C5, CFB, CFD, or CFH. After the intravenous administration of GSK933776 at 3, 6, or 15 mg/kg monthly for 18 months, plasma drug concentrations increased proportionally to the dose. After the increase in the GSK933776 concentration in plasma, plasma-free amyloid β levels declined rapidly at all doses, with near maximum reductions at the 15-mg/kg dose [74]. However, the intravenous administration of GSK933776 at 3, 6, or 15 mg/kg monthly for 18 months failed to slow GA progression or improve visual outcomes compared with placebo. Furthermore, pharmacogenetic analyses revealed no association between CFI gene polymorphisms and GA progression or differential response to GSK933776. The lack of treatment effect was attributed to several factors: (i) removing amyloid β does not modify disease progression in late AMD with GA, (ii) the stage of AMD treated may have been too advanced, (iii) cell death due to the enlargement of GA may have precluded detectable structural or functional preservation within an 18-month period.

#### 3.2.9. Emerging and Novel Complement Targets

Beyond selective C3 and C5 blockade, several alternative complement and upstream therapeutic strategies have been investigated in patients with GA, including C1q inhibition (ANX007), CFI or CFH restoration (GT005, VOY-101), properdin neutralization (CLG561, RO7303359), dual C3/C5 suprachoroidal inhibition (VV-14295), indirect oral modulation (EG-301), and Siglec-mediated macrophage and complement regulation (AVD-104) (Table S5). Although these therapies have progressed to clinical stages, no peer-reviewed publications reporting their clinical results are available to date. According to registry and sponsor-reported information, C1q blockade with ANX007 has progressed furthest among these candidates, advancing to Phase 3 trials following neuroprotective signals in Phase 2. In contrast, strategies such as GSK933776, CLG561, and GT005 were discontinued due to a lack of anatomical efficacy, while novel pipeline candidates, including AVD-104, VOY-101, VV-14295, and EG-301, remain under active early- to mid-stage clinical evaluation. As these results are not yet supported by peer-reviewed publications, they were not included in the evidence synthesis and are reported here for context only.

### 3.3. Quality Assessment

The results of the quality assessment with NOS are illustrated in Table S4. All studies reached an NOS score equal to or higher than 5. Among the 28 studies evaluated with NOS, five achieved a score of 9, six a score of 8, seven a score of 7, seven a score of 6, and three a score of 5.

Only three articles focused exclusively on PK, one [53] showing a population PK model of lampalizumab and two showing a population PK model [71] and a pharmacokinetic/pharmacodynamic analysis [31] of pegcetacoplan, all of them involving patients with GA. These studies were assessed using the ClinPK checklist. The overall compliance rate was 73%, and the main penalization was due to the use of data derived from previous clinical trials without providing a complete description of patient characteristics, insufficient detail regarding inclusion and exclusion criteria, lack of clear reporting of withdrawals, and absence of validation data of analytical techniques. Table S3 presents the individual scores obtained for each of the three studies.

## 4. Discussion

### 4.1. Current Landscape of Complement-Targeted Therapies for Geographic Atrophy

In order to synthesize the current landscape of complement-targeted therapies for GA, we carried out a systematic review by using PubMed, Cochrane and Embase databases. This systematic review provides an updated review of the therapeutic strategies for GA through modulation of the complement cascade, encompassing both marketed drugs and agents under clinical investigation. Despite the high prevalence of GA [76,77], treatment options besides conservative measures such as lifestyle modification (e.g., smoking cessation, exercise) and dietary supplementation remain limited [4]. In recent years, complement dysregulation has become a central focus in AMD pathogenesis, driving the development of several novel therapeutic candidates for GA [21].

Complement modulation for GA has been investigated at multiple levels of the complement cascade, although the available evidence varies considerably in terms of study design and clinical development stage. Upstream approaches have included recombinant CFH (GEM103) and inhibitors of CFB (sefaxersen/IONIS-FB-LRx) and CFD (lampalizumab, formerly FCFD4514S), whereas other strategies have targeted C3 (pegcetacoplan and NGM621) or C5 (avacincaptad pegol). Additional strategies include gene therapy with JNJ-81201887 (an AAV2 vector expressing soluble CD59) and GSK933776, an indirect strategy that acts upstream of the cascade by targeting a complement-modulating drusen constituent instead of a complement protein itself. Safety and tolerability have been evaluated in early-phase dose escalation studies for several investigational agents, including GEM103, sefaxersen, lampalizumab, and NGM621, as well as in randomized clinical trials for agents that progressed to later stages of development. Clinical efficacy in patients with GA has primarily been assessed in randomized controlled trials. To date, only pegcetacoplan and avacincaptad pegol have demonstrated a significant reduction in the rate of GA lesion growth, although this effect has not been consistently accompanied by improvements in visual function. Among the complement-targeted approaches investigated to date, only these two molecules have received regulatory approval, by both the FDA [37,39] and the TGA [38,40]. Additional post hoc analyses have provided further information on treatment efficacy and disease progression, while a real-world comparative study has also reported clinical outcomes for pegcetacoplan and avacincaptad pegol. Importantly, these post hoc and real-world findings should be interpreted as complementary to, rather than equivalent to, the evidence generated by randomized controlled trials.

As mentioned above, across several clinical trials involving numerous patients (Table 4 and Table 5, Table S5), pegcetacoplan and avacincaptad pegol have been shown to reduce the rate of GA lesion growth. In a 12-month retrospective real-world comparison between pegcetacoplan and avacincaptad pegol, both agents achieved comparable visual and anatomical outcomes, though pegcetacoplan required significantly fewer intravitreal injections [72], consistent with its broader FDA-approved dosing interval (every 29–60 days vs. every 4 weeks for avacincaptad pegol). These results are consistent with two recent syntheses of randomized controlled trials, a network meta-analysis of controlled trials involving different complement inhibitors [41], and a systematic review and meta-analysis restricted to C3/C5 inhibitor comparisons [42], which concluded that both avacincaptad pegol and pegcetacoplan significantly reduced GA growth relative to sham, measured as the change in square-root GA area at 12 months and in untransformed GA area at 24 months; moreover, direct and indirect comparisons of all interventions did not reveal significant differences in serious side effects. Contrary to this systematic review, these prior syntheses were restricted to complement inhibitors and did not address other complement targets under investigation, nor did they evaluate pharmacokinetic evidence or the emerging advanced drug delivery and gene-based strategies that may help overcome the limitations of currently approved therapies. A primary safety concern for both drugs is the increased risk of conversion to eAMD, with monthly pegcetacoplan demonstrating the highest incidence of macular neovascularization [42]. The association between neovascularization and complement inhibition is still poorly understood, but several theories have been proposed, including unmasking pre-existing subclinical neovascularization, or an elevated risk caused by surviving cells continuously producing VEGF in eyes with GA [18,61]. The conversion to eAMD has also been hypothesized to occur because the inhibition of the complement system may limit phagocytosis of the choriocapillaris endothelium by preventing deposition of complement components, allowing growth of new, leaky vessels lacking the barrier function of the mature endothelium [60].

Unfortunately, the delay in GA growth rate after the treatment with avacincaptad pegol or pegcetacoplan has not been consistently accompanied by a demonstrable improvement in visual function. This anatomical–functional dissociation deserves closer scrutiny, since the endpoints used across these trials are related but not interchangeable. Lesion area and growth rate (by autofluorescence or color photography), iRORA-to-cRORA progression, and OCT-based photoreceptor and ellipsoid-zone measures are structural surrogates, whereas BCVA, low-luminance BCVA, reading performance, retinal sensitivity, and contrast sensitivity are distinct clinical functional endpoints. None of these measures, taken alone, fully captures the impact of the disease on daily functioning. An ethnographic study of patients living with GA found substantial impairment in daily activities, particularly reading and driving, that did not correspond closely to BCVA level [76], supporting a European expert consensus call to weight patient-centered and functional outcomes alongside anatomical endpoints [77]. Framed as a distinction between the anatomical “lifespan” of a lesion and the functional “health span” of vision, this gap suggests that lesion growth rate, while a convenient trial endpoint, remains an incomplete proxy for clinically meaningful benefit [78]. Both approved agents require monthly to bimonthly IVT injections indefinitely, since the therapeutic effect decreases after treatment discontinuation; this treatment burden, together with the costs and risks inherent to repeated intraocular procedures, constitutes one of the most pressing unmet needs identified by patients, caregivers and clinicians. In fact, the refusal to authorize pegcetacoplan by the EMA, and the withdrawal of the avacincaptad pegol application both reflected concerns that the magnitude of anatomical benefit did not clearly offset the risks of chronic IVT dosing. Additional long-term studies to evaluate the impact of the treatment of patients with these two novel drugs on visual function and other metrics are needed to demonstrate if there is an actual benefit for quality of life [10,78].

A major hurdle in the clinical development of complement inhibitors lies in balancing drug exposure, potency, and administration burden. Standard IVT injections are restricted by a maximum tolerated volume, typically 100 µL. This limitation presents relevant formulation challenges, as seen for the anti-C3 monoclonal antibody NGM621, with 15 mg as the maximal deliverable dose per injection [34]. Similarly, C5-targeted drugs faced similar constraints; delivering higher doses of avacincaptad pegol (e.g., 4 mg) required split dosing, needing two separate 100 µL injections per treatment visit [55]. Sustained local complement suppression in chronic neuroretinal degeneration requires durable intraocular efficacy with less frequent dosing. Alternatively, systemic administration routes—including subcutaneous, intravenous, and oral modalities targeting upstream AP components such as the CFD inhibitor danicopan or CFB inhibitor iptacopan—have been explored. However, systemic complement blockade introduces off-target safety risks, systemic pharmacodynamic effects, and compliance challenges that contrast with localized ocular delivery.

Other drugs targeting the complement cascade have not demonstrated clinical efficacy despite strong preclinical and mechanistic rationales. Lampalizumab failed in two adequately powered Phase 3 trials [30], and NGM621 did not significantly reduce GA progression in the Phase 2 CATALINA trial (Table S5), whereas eculizumab [32] and GEM103 [28] were evaluated only in small pilot or Phase 1 studies that were not powered to detect efficacy, and the subsequent repeat-dose GEM103 trial was terminated (Table S5). The reasons for failure proposed by the investigators, such as dose limitations, suboptimal delivery routes, or intervention at a disease stage where complement no longer drives progression, highlight the challenge of translating biological plausibility into clinical benefit.

Despite strong genetic evidence implicating the complement system in AMD susceptibility and GA progression, no genetic variant has been validated as a predictive biomarker to identify patients most likely to respond to a specific complement-targeted therapy. This gap remains a key target for future trial designs, including the prespecified genetic stratification in ongoing early-AMD trials. For instance, in the FILLY trial [79], common risk variants in CFI, CFH, C2/CFB and C3 did not modify the treatment effect of pegcetacoplan, indicating that these loci were not predictive biomarkers for C3 inhibition. However, the C3 variant rs2230199 and the ARMS2 variant rs3750846 were associated with the underlying rate of GA lesion growth irrespective of treatment allocation, a prognostic association consistent with independent genome-wide association data. In the case of lampalizumab, in the Phase 2 MAHALO trial [52], it was suggested that carriers of a CFI risk-allele profile, defined jointly by variants in CFI, CFH and C2/CFB, derived substantially greater benefit from monthly treatment than the overall study population. Nevertheless, this predictive signal was not confirmed in the Phase 3 CHROMA and SPECTRI trials [30], where no significant difference in treatment effect was observed between CFI profile-positive and CFI profile-negative subgroups.

Beyond genetic biomarkers, demographic characteristics such as age and gender have also been explored as potential modifiers of treatment response, though far less consistently across the included studies. Mean age of the GA patients included in the identified studies ranged from 74.7 to 81.5 years (excluding the sefaxersen Phase 1 study, which was conducted in healthy volunteers with a mean age of 41–46 years). The proportion of female participants ranged from 40% to 75%, with the lowest value drawn from the study with NGM621 (Phase 1, n = 15) and the highest from the study with GEM103 (Phase 1, n = 12). Three studies formally analyzed age and/or gender in relation to disease progression or treatment effect: the MAHALO trial with lampalizumab [52], which found comparable treatment benefit between men and women within the CFI risk-allele carrier subgroup; a 12-month post hoc subgroup analysis of GATHER2 with avacincaptad pegol [57], which found the treatment effect to be consistent across demographic and baseline subgroups, including sex; and a post hoc analysis of the FILLY trial with pegcetacoplan [62], in which age and gender were evaluated as candidate risk factors for GA progression but—unlike extrafoveal lesion location, baseline visual acuity, and low-luminance deficit—were not identified as significant contributors. Taken together, the available evidence does not suggest a strong influence of age or gender on treatment response, although the small number of studies formally addressing this question and the differing analytical approaches used (risk factor analysis vs. treatment effect subgroup analysis) limit the strength of this conclusion.

Regarding pharmacokinetics, data have been only reported for lampalizumab [51,52,53], eculizumab [32], NGM621 [34], pegcetacoplan [31,71], and GSK933776 [74], and in most cases, only serum concentrations were measured, and ocular concentrations were predicted through PK and PK/PD models. Overall, the available PK data, which are largely based on serum measurements and model-based predictions of ocular exposure, indicate that IVT administration achieves sustained intraocular exposure with minimal systemic exposure, supporting the rationale for local complement inhibition while minimizing the likelihood of systemic complement-related adverse events. In only three studies was the drug not administered by the IVT route: sefaxersen was administered by subcutaneous injection to healthy volunteers, and eculizumab and GSK933776 were administered by intravenous infusion to patients. Intravenous administration of these two drugs failed to reduce the GA lesion growth rate. Although this outcome cannot be definitively attributed to low ocular drug levels, as intraocular concentrations were not quantified, from a pharmacological perspective, systemic drug administration is expected to result in limited drug exposure at the RPE when the target is to modulate local retinal complement activation, because the blood–retinal barrier (BRB) impedes the passage of large hydrophilic molecules into the retina [80]. The physicochemical and pharmacokinetic properties of the molecules assayed in clinical trials may be responsible, at least in part, for the limited efficacy achieved. In fact, all agents that reached clinical testing for GA are large (molecular weights ranging from approximately 10 to 150 kDa), hydrophilic biologics, i.e., monoclonal antibodies or antibody fragments (lampalizumab, NGM621, eculizumab, GSK933776), a PEGylated peptide (pegcetacoplan), or a PEGylated RNA aptamer (avacincaptad pegol). Because these molecules must instead be delivered directly into the vitreous, their clinical performance is dictated as much by ocular pharmacokinetics as by target biology. On the one hand, the need for IVT dosing implies, in turn, pharmaceutical challenges and delivery limitations: the maximum volume that can be safely injected intravitreally (~100 µL) imposes a hard ceiling on deliverable dose, which required NGM621 to be capped at 15 mg [34] and avacincaptad pegol 4 mg to be split into two separate 2 mg injections [55]; on the other hand, IVT half-life determines whether monthly re-dosing is required to sustain target engagement, as modelled for lampalizumab [53] and pegcetacoplan [31,71].

Beyond the approved therapies and published clinical investigations, numerous clinical trials targeting the complement cascade are ongoing or have concluded without reported peer-reviewed results (Table S5). These include tesidolumab/LFG316 (C5 inhibitor, NCT01527500), POT-4/AL-78898A (C3 inhibitor, NCT01603043), ARC1905 (C5 inhibitor, NCT00709527), and IONIS-FB-LRx (CFB inhibitor, NCT03815825), among others. This sustained clinical activity (up to 31 clinical trials) reflects ongoing industry interest in developing novel therapeutics for GA.

A question of direct practical relevance is whether one specific node of the complement cascade, C3, C5, or a still more upstream target is mechanistically superior. The evidence assembled in this review does not support a definitive conclusion. Considering the clinical experience with pegcetacoplan and avacincaptad pegol, neither C3 nor C5 inhibition has been convincingly shown to be superior over the other regarding clinical outcomes; therapeutic choice in clinical practice is currently guided more by safety profiles, dosing schedules, and clinical experience than by a demonstrated mechanistic advantage. From a mechanistic perspective, arguments favor both targets: C5 inhibition acts downstream, leaving upstream C3-derived fragments (such as iC3b) available to facilitate opsonization and clearance of cellular debris and drusen material, functions essential for retinal homeostasis [81]. Separately, RPE cells have been shown, in an ex vivo cell culture system, to mitigate complement attack through endocytic clearance of C5b-9 [82], indicating an intrinsic cellular defense mechanism against MAC-mediated injury, though this observation does not by itself establish therapeutic efficacy for any complement-targeted intervention. Conversely, C3 inhibition blocks the cascade earlier and more completely, suppressing the generation of both C3a and C5a anaphylatoxins as well as MAC formation [83], which provides broader anti-inflammatory coverage but risks interfering more extensively with physiological ocular functions. Targets positioned further upstream—CFB, CFD and CFH—were designed to achieve selective alternative pathway inhibition while sparing the classical and lectin pathways; however, this theoretical selectivity has not yet translated into clinical benefit: lampalizumab failed in Phase 3 trials [30], the repeat-dose GEM103 trial was terminated (Table S5), and peer-reviewed data for sefaxersen are limited to healthy volunteers [29]. Taken together, the evidence reviewed here suggests (although it does not establish) that the precise node of inhibition may matter less than achieving adequate and sustained target engagement. In this regard, drug delivery strategies and pharmacokinetic profiles are critical determinants for developing effective and safe therapies for GA.

Given the persisting uncertainty regarding optimal single-target inhibition, and considering that complement activation converges from three initiating pathways, other rational strategies for durable disease control include interrupting the alternative-pathway amplification loop, combining multi-node complement inhibition, or pairing complement blockade with independent therapeutic pathways. An earlier proof-of-concept study evaluated the combination of an anti-properdin Fab fragment (CLG561) (targeting properdin, a positive regulator of the alternative pathway) with the anti-C5 antibody LFG316 (tesidolumab) to test whether simultaneous alternative and terminal-pathway blockade improved efficacy relative to monotherapy (NCT02515942) [84]. This randomized, sham-controlled, Phase 2 proof-of-concept trial was completed in 2017; however, no peer-reviewed publication reporting its results is currently available. More recently, an ongoing Phase 3 trial (SIENNA, NCT06541704) [85,86] is evaluating cemdisiran, a small interfering RNA (siRNA) that reduces hepatic synthesis of C5, administered by subcutaneous route either alone or in combination with pozelimab, an anti-C5 monoclonal antibody, with the goal of achieving near-complete and sustained suppression of C5 activity. This trial is currently recruiting, with an estimated primary completion date in 2027, and no efficacy or safety results have yet been reported. As neither combination strategy is supported by peer-reviewed clinical data, both are presented here as investigational approaches derived from registry information, and are not considered evidence of comparable strength to the peer-reviewed randomized trials discussed elsewhere in this review. The absence of published outcomes is acknowledged as a limitation of the current evidence base for combined complement inhibition.

### 4.2. Advanced Drug Delivery Systems, Nanotechnology-Based Carriers and Gene Therapy

Following the systematic review, we analyzed the emerging therapeutic strategies that may help overcome the current limitations of complement-targeted therapies and improve their clinical performance, including advanced drug delivery systems and gene-based approaches.

Since treatment burden, rather than lack of biological rationale, represents one of the principal barriers limiting the clinical impact of complement inhibitors, advanced drug delivery technologies represent a logical and pharmaceutically relevant avenue for improvement, as reviewed for AMD more broadly [87]. Sustained-release IVT implants have already been validated clinically for other posterior-segment diseases, for example, the non-biodegradable fluocinolone acetonide implants Retisert^®^ and Iluvien^®^ [88]. Biodegradable platforms have also been explored preclinically for other ocular drugs: for instance, a cynomolgus monkey PK study of a biodegradable brimonidine (a selective α2-adrenoceptor agonist) posterior-segment delivery implant combined with translational PK modeling predicted that a single intraocular procedure could maintain therapeutic drug concentrations in the human retina for several months instead of weeks [89]. Similarly, refillable port-delivery platforms (a hollow drug reservoir composed of a nonbiodegradable polysulfone body coated in silicone, which is inserted into the pars plana and anchored within the sclera by an extrascleral flange and allows for the sustained delivery of the drug) have been clinically evaluated for anti-VEGF agents in neovascular AMD, where office-based refill procedures have been shown to replace the large majority of IVT injections while preserving clinical efficacy [90,91]. The ranibizumab port-delivery system [90,91] is an example of an established, clinically evaluated ocular delivery platform, but its applicability to other agents, including complement modulators, has not yet been clinically tested.

Nanotechnology-based carriers offer additional opportunities beyond simple sustained drug release. Polymeric and lipid nanoparticles (NPs), hydrogels, dendrimers, and nanoceria (cerium oxide nanoparticles) have been proposed as platforms capable of prolonging IVT residence time, protecting labile biologics from degradation, and, in certain formulations, conferring additional biological activity [92]. Although there have been significant advancements in the use of diverse NPs for AMD in vitro, in vivo, and ex vivo, a deeper understanding of NP–biological interactions is still essential to translate these technologies from bench to bedside. In fact, no peer-reviewed clinical efficacy data in GA have been reported to date. An illustrative example is provided by sialic acid- and polysialic acid-decorated NPs (PolySia-NPs, ~100 nm), composed of a PEG-PLGA core covalently attached to polysialic acid that selectively binds human Siglec receptors, designed to modulate macrophage- and complement-driven inflammation in GA/AMD. This candidate has been characterized in a dedicated preclinical safety study. The study included in vitro genotoxicity assays, systemic toxicity testing after intravenous administration in rodents, and ocular/systemic toxicity assessment after IVT administration in rabbits and non-human primates, with no adverse clinical or ophthalmological findings reported [93]. These preclinical findings support further development; this platform has since entered clinical evaluation as AVD-104 (Section 3.2.9; Table S5), but no peer-reviewed clinical efficacy data in GA are yet available.

As with the sustained-release implants and port-delivery platforms described above, the nanoparticle and gene-vector-based strategies are conceived as intraocular delivery systems rather than systemic formulations. Systemic administration of these carriers would not achieve adequate local concentration and would additionally increase off-target biodistribution and immunogenicity risk. Their intended clinical benefit is therefore not a less invasive route of administration, but a reduction in dosing frequency, by prolonging intraocular residence time. Additionally, these systems may offer superior protection against degradation for labile cargo compared to the intraocular injection solutions used in clinical trials.

Advanced computational approaches could provide a useful framework through which to address remaining formulation hurdles in nanoparticle-based therapies for dry AMD. By analyzing high-dimensional datasets, machine learning algorithms can map complex relationships between nanoparticle physicochemical properties, in vivo pharmacokinetic behavior, and biological efficacy. For instance, by modeling how nanoparticle hydrodynamic dimensions, zeta potential and surface modifications govern vitreoretinal transport and clearance, these computational models could help establish a predictive framework to optimize nanoparticle engineering. Furthermore, machine learning-driven high-throughput screening could accelerate the identification of optimal drug nanocarrier formulations tailored to specific disease phenotypes, which may ultimately support precision therapy for GA [92], although their application to complement-targeted delivery systems for GA remains to be demonstrated.

Gene therapy represents a paradigm shift from repeated exogenous protein delivery dosing toward sustained intraocular synthesis of the therapeutic agent, potentially reducing the chronic treatment burden. The primary goal of gene therapy is long-term expression of a transgene at therapeutic levels (supplementation gene therapy), though suppression of pathological genes can also be achieved using RNA interference or genome-editing tools [94] A foundational milestone in ocular gene therapy was the clinical approval of voretigene neparvovec (Luxturna), an AAV-based gene therapy indicated for RPE65-mediated inherited retinal dystrophies, approved by both the FDA and the EMA. Luxturna served as a clinical proof of concept, establishing the feasibility, ocular safety, and therapeutic potential of targeted genetic interventions for retinal diseases [95,96].

In neovascular AMD, gene therapy aims to provide continuous intraocular anti-VEGF expression to reduce injection frequency, whereas in dry AMD/GA, the purpose is to retard tissue atrophy by modulating complement activation as discussed in recent narrative reviews of gene therapy for AMD and GA [97,98]. One example is JNJ-81201887 [35], a recombinant AAV2 vector expressing a soluble form of CD59 to inhibit MAC formation regardless of the initiating complement pathway. In preclinical animal models, AAV2-mediated sCD59 expression after IVT administration significantly reduced MAC deposition [99]. Separately, in a Phase 1 human clinical trial in patients with GA (NCT03144999), the treatment was well tolerated, but its potential efficacy could not be established given the study’s safety-focused design [35]. Another example is GT005, an AAV2 vector encoding CFI, the primary negative regulator of C3b. Administered as a single subretinal injection, GT005 was designed to provide sustained CFI overexpression, thereby continuously restraining alternative pathway amplification. Although evaluated across multiple clinical trials in GA secondary to AMD (NCT03846193, NCT05481827, NCT04437368, NCT04566445), the development program was terminated because futility criteria were met and the overall benefit–risk ratio did not support continuation (Table S5). Together, these two examples illustrate the potential of gene therapy to reduce the need for chronic IVT injections, while underscoring that neither candidate has yet provided peer-reviewed evidence of durable clinical efficacy in GA.

Although AAV vectors remain the preferred platform in ocular gene therapy due to their favorable safety profile, broad retinal tropism, and capacity for long-term transgene expression after a single administration [100], alternative viral platforms such as lentiviral vectors are under investigation. While lentiviral vectors can accommodate larger genetic payloads, their clinical translation remains constrained by suboptimal retinal cell tropism and potential safety risks associated with genomic integration, as discussed in a general (non-retina-specific) review of lentiviral vector engineering [101]. Despite substantial advances in vector engineering, clinical application of viral gene therapy products faces persistent challenges regarding immunogenicity and toxicity. Additional manufacturing hurdles include high production costs, complex scale-up, vector potency standardization, and strict cold-chain requirements (e.g., deep-freeze storage, precise clinical dilution protocols, and narrow post-thaw usability windows) [102]. These challenges have been described in the context of viral vector manufacturing generally, not specifically for ocular products.

As an alternative to viral vectors, non-viral delivery platforms (including lipidic, polymeric, dendrimers, and inorganic systems, among others) offer distinct advantages in safety, cost, scalable manufacturing, low immunogenicity, repeated-dosing capability, large payloads, and flexibility of design. However, regardless of the target disease, they typically induce lower and more transient transgene expression compared to AAV systems [103]. Advances in materials science and nanotechnology have significantly accelerated the development of non-viral vectors for both ocular and non-ocular diseases [104]. In the specific field of ocular applications, lipid-based nanoparticles have demonstrated the ability to mediate nucleic acid delivery to retinal tissues in preclinical models; however, their application has not yet been specifically investigated in GA or complement-mediated disease models. A recent study has shown that mRNA encoding GFP and ciliary neurotrophic factor (CNTF) formulated in LNP were able to transfect mouse eye tissues in vivo (aged male CD-1 mice were injected intravitreally with the mRNA LNPs), and human RPE and retinal explants [105]. Although this study did not specifically use GA models, it showed that IVT administration of mRNA-LNPs could transfect the RPE in mice. In addition to LNPs, solid lipid nanoparticles (SLNs) are a promising gene delivery platform. Their surface can be functionalized with targeting ligands to facilitate selective cellular uptake, influence intracellular trafficking, and modulate nucleic acid release, potentially improving both therapeutic efficacy and safety [106]. Cationic SLN-based vectors have also been shown to transfect multiple retinal layers following single IVT or subretinal injection in a mouse model of X-linked juvenile retinoschisis, a retinal degenerative disorder caused by mutations in the *RS1* gene encoding retinoschisin [107,108]. SLNs successfully delivered sufficient retinoschisin to partially repair the damaged retinal structure in the animal subjects. Moreover, our research group has demonstrated that SLNs are suitable carriers for delivering different types of nucleic acids, including plasmid DNA and mRNA [109]. Although these studies were not conducted in the context of GA or the complement cascade, they provide evidence supporting the potential of SLNs as versatile delivery platforms for gene therapy applications.

As mentioned above, targeting multiple complement nodes or combining complement blockade with independent neuroprotective pathways may yield more comprehensive disease control than single-target monotherapy. Nanotechnology and non-viral gene therapy offer unique opportunities to engineer co-delivery platforms capable of overexpressing or downregulating more than one complement cascade element, or pairing complement inhibition with a mechanistically independent, non-complement pathway (for instance, neuroprotective molecules, such as CNTF or glial cell line-derived neurotrophic factor (GDNF), which contribute to photoreceptor survival under oxidative and inflammatory stress) [110]. While accommodating large genetic payloads within a single AAV vector remains restrictive, non-viral delivery platforms provide a much higher packaging capacity. For example, we developed a hybrid nanomedicine based on SLN and gold nanoparticles (“golden SLNs”) for the co-delivery of a *GLA* plasmid and an siRNA against globotriaosylceramide (Gb3) synthase to simultaneously promote α-Galactosidase A production and inhibit the synthesis of Gb3 as a dual therapeutic strategy for Fabry disease [111]. This co-delivery vector effectively protected both nucleic acids from nuclease-mediated degradation while promoting efficient cellular internalization and endosomal escape. These findings highlight the potential of lipid-based nanoplatforms for the simultaneous delivery of multiple nucleic acids, which could be relevant to GA by enabling the targeting of more than one complement modulator. Whether this holds in GA-relevant models has yet to be confirmed.

It should be emphasized that no non-viral, nanoparticle-based gene therapy specifically targeting the complement cascade has yet reached clinical trials for GA; the evidence summarized herein derives from preclinical AMD models and other retinal disease indications. Nonetheless, these studies support the feasibility of these platforms and point to specific opportunities that could complement or eventually compete with AAV-based approaches for GA.

### 4.3. Strengths and Limitations of This Review

This review has several strengths, including a comprehensive search of three databases complemented by manual searching, a structured quality assessment of the included clinical studies using the NOS, and an assessment of reporting completeness for pharmacokinetic studies using the ClinPK checklist. Some limitations should be acknowledged. First, although the search strategy combined controlled vocabulary with free-text terms for the disease and mechanistic concepts and was supplemented by manual searching of reference lists and screening of clinical trial registries, we cannot fully exclude the possibility that records indexed exclusively under individual drug or developmental code names, without co-occurring disease or complement terminology, were missed; nevertheless, this is considered unlikely given that all agents identified as complement-targeting therapies for GA are expected to co-index with both disease and mechanistic terms. Second, heterogeneity of study designs, outcome definitions and follow-up durations across the included trials limited the possibility of comparative synthesis. Third, several relevant studies, including recently reported nanoparticle- and gene-based candidates, were identified through the narrative discussion rather than the systematic search itself, since eligible peer-reviewed clinical data in humans were not yet available for these strategies at the time the search was conducted. Finally, restricting the systematic component of the review to human studies, while methodologically appropriate for the primary objective, excluded the substantial preclinical body of evidence that continues to inform the rational design of next-generation drug delivery and gene therapy platforms for GA.

## 5. Conclusions

The clinical evidence synthesized in this systematic review shows that only C3 inhibition with pegcetacoplan and C5 inhibition with avacincaptad pegol have significantly slowed GA lesion growth in randomized pivotal trials and that this effect is modest (approximately 14–29% versus sham) and variable across agents, doses, regimens, and endpoints. Neither agent improved BCVA or low-luminance BCVA, and both increased the incidence of exudative AMD. Post hoc analyses suggesting structural and functional benefits are hypothesis-generating and require prospective confirmation. Strategies targeting upstream complement components, such as CFB, CFD and CFH, have so far failed to translate into clinical benefit, and the requirement for frequent intraocular administration adds a substantial treatment burden. The available pharmacokinetic data, largely model-based, indicate that the intravitreal half-life of the active molecules and the injection volume limits require frequent intraocular administration.

Gene therapy and advanced delivery systems continue to show experimental promise rather than demonstrated clinical benefit in GA. Clinical experience is confined to two AAV2-based vectors: JNJ-81201887 as evaluated in a Phase 1 safety study and GT005 whose development program was terminated because the overall benefit–risk ratio did not support continuation. Moreover, sustained-release implants and port-delivery systems have been validated only for other drugs, and no nanotechnology-based carrier has yet reported peer-reviewed clinical efficacy data in GA. Their translation will require evaluation of immunogenicity, long-term safety, manufacturing consistency, and treatment durability, as well as of the long-term consequences of sustained complement modulation, including infection and neovascular risk. Further preclinical and clinical studies are needed to establish whether these approaches can provide durable, multitarget, and patient-centered management of GA.

## Figures and Tables

**Table 1:** Characteristics of the study involving recombinant complement factor H.

Drug (API) Developer	Author, Year, Ref. No.	Clinical Trial ID, Phase, Design	Administration Route, Dose, Duration	Subjects (*n*), Follow Up	Outcome Description	Mean Clinical Outcomes
GEM103 (recombinant human CFH) Gemini Therapeutics Inc., Cambridge, MA, USA	Khanani et al. Am et al., 2022 [28]	NCT04246866 Phase I Single-ascending-dose open-label clinical trial.	IVT 50, 100, 250, 500 µg/eye; single injection.	12 patients. Mean age: 81.5 years Female: 75% Follow up: 2 months (50, 100, 250 µg/eye) 4 months (500 µg/eye).	Safety assessments: ocular and systemic adverse events, ocular examinations, clinical laboratory and vital signs, and serum antidrug antibody levels. Biomarkers, measured in aqueous humour: CFH, factor Ba and C3a.	GEM103 was well tolerated. No dose-limiting toxicity reported. BCVA and LLBCVA stable throughout the study. Increased aqueous humour CFH levels up to 8 weeks post-injection at doses ≥ 100 µg/eye.

IVT: intravitreal, CFH: human complement factor H, BCVA: best corrected visual acuity, LLBCVA: low-luminance best corrected visual acuity.

**Table 2:** Characteristics of the studies involving silencing of complement factor B.

Drug (API), Developer	Author, Year, Ref. No.	Clinical Trial ID, Phase, Design	Administration Route, Dose, Duration	Subjects (*n*), Follow Up	Outcome Description	Mean Clinical Outcomes
Sefaxersen (IONIS-FB-LRx, antisense inhibitor of CFB) Iveric Bio (Astellas Pharma, Tokyo, Japan)	McCaleb ML, et al. 2025 [29]	EudraCT number 2015–001837- 25 (SAD) ACTRN12616000335493 (MAD) Randomized, placebo-controlled, double-blind, single-ascending-dose phase 1 study.	SC SAD: 10, 20, 40 mg MAD: 10, 20 mg/week during 6 weeks (4 loading doses the first 2 weeks and once-a-week for the next 4 weeks).	Healthy volunteers SAD: 24 Mean age 41 years Female: 12% MAD: 30 Mean age: 46 years Female: 27% Follow up: 90 days.	Safety and tolerability. Blood chemistries, routine hematology, coagulation parameters and urinalysis. Vital signs, ECG, physical examination and concomitant medication usage. Plasma quantification of CFB, AP, CP activity, Bb, C2, C4, CFD.	Sefaxersen was well tolerated and effectively reduced systemic CFB and alternative pathway activity.

MADs: multiple ascending doses, SAD: single ascending dose, SC: subcutaneous. ECG: electrocardiogram, CFB: complement factor B, AP: alternative pathway, CP: classical pathway, Bb: Bb fragment of complement factor B, C2: complement component 2, C4: complement component 4, CFD: complement factor D.

**Table 3:** Characteristics of the studies involving complement factor D inhibitors.

Drug (API), Developer	Author, Year, Ref. No.	Clinical Trial ID, Phase, Design	Administration Route, Dose, Duration	Subjects (*n*), Follow Up	Outcome Description	Mean Clinical Outcomes
FCFD4514S (Lampalizumab, CFD monoclonal antibody fragment) Genetech, Inc., South San Francisco, CA, USA.	Do DV, et al. 2014 [51]	NCT00973011 Multicenter, open-label, single-dose, dose-escalation Phase 1a study.	IVT Single dose (0.1, 0.5, 1, 2, 5 and 10 mg)	18 patients Mean age: 74.7 years Female: 67% Follow up: 90 days.	Primary outcome: safety and tolerability of the drug. Secondary outcome: characterization of serum pharmacokinetics (AUC, Cmax, Tmax, t_1/2_).	FCFD4514S was well tolerated with no dose-limiting toxicity or immunogenicity, and low systemic exposure that declined over time up to the maximum tolerated dose of 10 mg.
FCFD4514S (Lampalizumab, CFD monoclonal antibody fragment) Genentech, Inc., South San Francisco, CA, USA.	KN Le, et al. 2015 [53]	NCT00973011: multicenter, open-label, single-dose, dose-escalation, Phase 1a study. NCT01229215 (MAHALO Phase 1b/2, unpublished): multicenter, randomized, single-masked, Phase 2 study.	IVT NCT00973011: 0.1 to 10 mg, single dose NCT01229215: 3 monthly doses of 10 mg 10 mg every month or 10 mg EOM 18 months	117 patients (serum pharmacokinetics) 21 patients (ocular pharmacokinetics) No separate follow-up period reported.	PK data: ocular and systemic lampalizumab concentrations, CFD activity, and PK/PD parameters.	Population PK/PD modelling showed that IVT lampalizumab achieves high ocular concentrations with low systemic exposure and predicts near-complete target engagement in the vitreous for proposed dosing regimens.
FCFD4514S (Lampalizumab, CFD monoclonal antibody fragment) Genentech, Inc., South San Francisco, CA, USA.	Yaspan BL, et al. 2017 [52]	NCT01229215 (MAHALO) multicenter, randomized, single-masked, Phase 2 study.	IVT 10 mg monthly or EOM during 18 months	123 patients Mean age: 78.7 years Female: 56.9% 18 months 17/99 patients: continued 3-month safety follow-up 82/99: continued into an open-label extension study.	Primary efficacy outcome: mean change in GA area by FAF. Secondary outcome: mean change in GA area by colour fundus photography, and mean change in BCVA (ETDRS). Safety: incidence and severity of ocular and systemic adverse events, changes and abnormalities in clinical laboratory parameters, and incidence of positive serum antibodies to lampalizumab.	Monthly lampalizumab reduced GA lesion area progression by 20% versus sham In complement factor I risk-allele carriers, 44% reduction in GA lesion area progression. Accepted safety profile.
FCFD4514S (Lampalizumab, CFD monoclonal antibody fragment) Hoffmann–La Roche, Basel, Switzerland	Holz FG, et al. 2018 [30]	NCT02247479 (Chroma) NCT02247531 (Spectri) Double-masked, multicenter, randomized, sham injection–controlled Phase 3 clinical trials.	IVT 10 mg every 4 or 6 weeks, 96 weeks	Chroma: 906 patients Spectri: 975 patients Mean age: 78.0 years Female: 60% No separate follow-up period reported.	Primary efficacy outcome: mean change in GA lesion area by FAF. Secondary efficacy outcomes: visual function were exploratory at week 48. Safety (adverse events, deaths, electrocardiograms, antidrug antibodies, ocular assessments).	Lampalizumab did not significantly reduce geographic atrophy progression versus sham, with no benefit observed in biomarker-defined subgroups.
FCFD4514S (Lampalizumab, Complement factor D monoclonal antibody fragment) Hoffmann–La Roche, Basel, Switzerland	Edmonds R, et al. 2023 [54]	NCT02247479 (Chroma) NCT02247531 (Spectri) Double-masked, multicenter, randomized, sham injection-controlled Phase 3 clinical trials	IVT 10 mg every 4 or 6 weeks for 96 weeks	97 patients Mean age 79.6 years Female: 63.9% No separate follow-up period reported.	Complement activity in aqueous humour.	Increased CFD level at week 24 compared with baseline. Median reduction in the Bb–CFB ratio of 41% to 43%. No strong correlations between lampalizumab concentrations in aqueous humour and change in CFD levels or Bb:CFB ratio over time.

CFD: complement factor D, IVT: intravitreal, AUC: area under the curve, Cmax: maximum observed serum concentration, Tmax: time of maximum observed concentration, T1/2: terminal half-life, CFB: human complement B, Bb: Bb fragment of CFB, PK/PD: pharmacokinetic/pharmacodynamic, EOM: every other month, GA: geographic atrophy, FAF: fundus autofluorescence, BCVA: best corrected visual acuity, ETDRS: Early Treatment of Diabetic Retinopathy Study.

**Table 4:** Characteristics of the studies involving C5 inhibitors.

Drug (API), Developer	Author, Year, Ref. No.	Clinical Trial ID, Phase, Design	Administration Route, Dose, Duration	Subjects (*n*), Follow Up	Outcome Description	Mean Clinical Outcomes
Eculizumab (complement C5 monoclonal antibody inhibitor) Alexion Pharmaceuticals, Boston, MA, USA.	Yehoshua Z, et al. 2014 [32]	NCT00935883 (COMPLETE) Prospective, sham controlled, double-masked, randomized clinical trial, Phase 2 study.	Intravenous infusion Low dose: 600 mg/week for 4 weeks followed by 900 mg/week every 2 weeks until week 24. High dose: 900 mg/week for 4 week followed by 1200 mg/week (high dose) every 2 week until week 24.	30 patients Mean age: 79 years Female: 70% Follow up: 26 weeks	Primary endpoint: Change in area of GA. Secondary endpoints: visual and low luminance visual acuities, choroidal thickness, PK, renal function and C-reactive protein levels.	Well tolerated. Lack of efficacy (no decrease in the growth rate of GA).
Avacincaptad pegol (PEGylated aptamer targeting complement C5) Iveric Bio (Astellas Pharma, Tokyo, Japan)	Jaffe GJ, et al. 2021 [55]	NCT02686658 (GATHER1) Prospective, randomized, double-masked, sham controlled, Phase 2/3 pivotal clinical trial.	IVT Part 1: 1 mg, 2 mg, monthly, 18 months Part 2: 2 mg, 4 mg, monthly, 18 months	286 patients. Part 1: 77. Part 2: 209 Mean age: 78.2 Female: 70.3% No separate follow-up period reported.	Primary efficacy endpoint: mean rate of change in GA over 12 months measured by FAF.	Well tolerated. Significant reduction in GA growth (27.4% in 12 months vs. sham).
Avacincaptad pegol (PEGylated aptamer targeting complement C5) Iveric Bio (Astellas Pharma, Tokyo, Japan)	Patel SS, et al. 2023. [56]	NCT02686658 (GATHER 1) Prospective, randomized, double-masked, sham-controlled, Phase 2/3 pivotal clinical trial.	IVT Part 1: 1 mg, 2 mg, monthly, 18 months Part 2: 2 mg, 4 mg, monthly, 18 months	286 patients. Part 1: 77 Part 2: 209 Mean age: 78.2 years Female: 70.3% No separate follow-up period reported.	Primary endpoint: GA lesion area by FAF Secondary endpoints: mean change BCVA low-luminance BCVA (LLBCVA; ETDRS letters). Safety endpoints.	Well tolerated. 2 and 4 mg showed continued reduction in the progression of growth lesion compared with sham.
Avacincaptad pegol (PEGylated aptamer targeting complement C5) Iveric Bio (Astellas Pharma, Tokyo, Japan)	Khanani AM, et al. 2023 [57]	NCT04435366 (GATHER2) Randomised, double-masked, sham controlled Phase 3 trial.	IVT Part 1: 2 mg monthly, 12 months. Part 2: 2 mg, monthly or EOM, 12 months	448 patients Mean age: 77 years Female: 68% No separate follow-up period reported.	Primary endpoint: GA lesion size by FAF. Secondary endpoints: change in BCVA and LL-BCVA.	Significantly slower mean rate of GA growth versus sham. No differences in mean change in BCVA or LL-BCVA between treatment groups.
Avacincaptad pegol (PEGylated aptamer targeting complement C5) Iveric Bio (Astellas Pharma, Tokyo, Japan)	Danzig CJ, et al. 2024. [58]	Post hoc NCT04435366 (GATHER2) NCT02686658, (GATHER1) Randomized, double-masked, sham-controlled Phase 3 trials.	IVT GATHER 1: Part 1: 1 mg or 2 mg monthly up to 12 months. Part 2: 2 mg or 4 mg monthly up to 12 months. GATHER 2: Part 1: 2 mg monthly (12 months). Part 2: 2 mg monthly or EOM (12 months)	624 patients Mean age: 77 years Female 68.2% No separate follow-up period reported.	Categorical Change in BCVA, reduction in relative risk in persistent vision loss, loss of BCVA to a level below driving eligibility.	Treatment was associated with small but statistically significant reduction in event rates of ≥15- and ≥20-letter BCVA ETDRS letter losses compared with sham.
Avacincaptad pegol (PEGylated aptamer targeting complement C5) Iveric Bio (Astellas Pharma, Tokyo, Japan)	Corradetti G, et al. 2025 [59]	Post hoc NCT02686658 (GATHER 1) Prospective, randomized, double-masked, sham-controlled Phase 2/3 study.	IVT Part 1: 1 mg or 2 mg monthly up to 18 months. Part 2: 2 mg or 4 mg monthly up to 18 months. Participants in the ACP 2 mg and sham groups were included in this analysis if they received at least one dose of study drug and had gradable baseline and follow-up images and the presence of iRORA and/or drusen of at least 40 μm in height at baseline.	286 patients Mean age: 77 years Female: 59% No separate follow-up period reported.	GA progression, iRORA to cRORA progression, Drusen to iRORA/cRORA progression.	Decreases of the proportion of eyes that progress from iRORA to cRORA and from drusen to iRORA or cRORA, compared with sham.
Avacincaptad pegol (PEGylated aptamer targeting complement C5) Iveric Bio (Astellas Pharma, Tokyo, Japan)	Khanani, AM et al. 2026 [33]	NCT04435366 (GATHER 2) Phase 3, randomized, sham-controlled study.	IVT 2 mg (n = 225) or sham (n = 222) for 1 year. At month 12, patients who received 2 mg were randomized again 1:1 to dosing every month or EOM with ACP 2 mg. Patients who had received monthly sham continued with sham.	447 patients Mean age: 77 years Female: 68% With 447 eyes of 447 patients randomized at baseline (225 eyes to monthly ACP 2 mg and 222 eyes to monthly sham administration), which included 1 eye randomized to the sham group that was not treated. Of the 189 patients assigned to the ACP 2 mg group who were randomized again after the primary analysis at year 1, 96 patients were assigned to ACP 2 mg every month, and 93 patients were assigned to ACP 2 mg EOM in year 2. Of the 222 patients initially assigned to re-randomized the sham group, 203 patients remained in the study at the end of year 1 and continued in the sham group in year 2.	The safety and efficacy of ACP versus sham administration over 2 years and the effect of ACP every month or EOM dosing in year 2.	ACP sustained a reduction in GA progression over 24 months, reducing lesion growth by 14% (monthly dosing) and 19% (EOM dosing) versus sham. Earlier treatment initiation provided greater cumulative anatomical benefit, although no significant improvements in BCVA or other visual function outcomes were observed.

IVT: intravitreal, GA: geographic atrophy, PK: pharmacokinetics, EOM: every other month, ACP: avacincaptad pegol, FAF: fundus autofluorescence, BCVA: best corrected visual acuity, LLBCVA: low-luminance best corrected visual acuity, ETDRS: Early Treatment of Diabetic Retinopathy Study, iRORA: incomplete retinal pigment epithelial and outer retinal atrophy, cRORA: complete retinal pigment epithelial and outer retinal atrophy.

**Table 6:** Characteristics of the study comparing pegcetacoplan and avacincaptad pegol.

Drug (API) Developer	Author, Year, Ref. No.	Clinical Trial ID, Phase, Design	Administration Route, Dose, Duration	Subjects (*n*), Follow Up	Outcome Description	Mean Clinical Outcomes
Avacincaptad pegol (PEGylated aptamer targeting complement C5) Pegcetacoplan (PEGylated peptide-targeting complement C3) Apellis Pharmaceuticals, Inc., Waltham, MA, USA (Pegcetacoplan) Iveric Bio (Astellas Pharma, Tokyo, Japan) (Avacincaptad Pegol)	Rush RB, et al. 2025 [72]	Retrospective, comparative assessment (“real word”)	IVT Pegacetacoplan: 15 mg, every 25 to 60 days Avacincaptad Pegol: 2 mg, every 4 weeks 12-months	112 Pegacetacoplan: 52 Avacincaptad Pegol: 60 Mean age 79.4 Female 50.6% No separate follow-up period reported.	Primary outcome: change in GA total surface area at the end of the month 12. Secondary outcomes: change in visual acuity at the end of the 12-month (study period).	Both treatments showed comparable GA progression and visual outcomes at 12 months. Pegacetacoplan required fewer injections to obtain this outcome.

GA: geographic atrophy, IVT: intravitreal, MNV: macular neovascularization.

**Table 7:** Characteristics of the study evaluating CD59-based therapies for geographic atrophy.

Drug (API), Developer	Author, Year, Ref. No.	Clinical Trial ID, Phase, Design	Administration Route, Dose, Duration	Patients (n), Follow Up	Outcome Description	Mean Clinical Outcomes
JNJ-81201887 (AAV2-based gene therapy expressing soluble CD59) Johnson & Johnson Company, New Brunswick, NJ, USA	Jeffrey S. Heier et al. 2024 [35]	NCT03144999 Phase 1, open-label, single-center, first-in-human clinical study.	IVT Single injection of low-dose (3.56 × 10^10^ viral genome/eye), intermediate-dose (1.07 × 10^11^ viral genome/eye), and high-dose (3.56 × 10^11^ viral genome/eye).	17 patients 64.7% female Follow up: 24 months	Safety and tolerability outcomes included assessment of ocular and non-ocular treatment-emergent adverse events over 24 months. Secondary outcomes included GA lesion size and growth rate.	A single IVT administration demonstrated an acceptable safety profile and sustained intraocular expression of sCD59 for up to 24 months.

GA: geographic atrophy, IVT: intravitreal, AAV2: adeno-associated virus serotype 2, sCD59: soluble CD59.

**Table 8:** Characteristics of the study involving an anti-amyloid β monoclonal antibody.

Drug (API), Developer	Author, Year, Ref. No.	Clinical Trial ID, Phase, Design	Administration Route, Dose, Duration	Subjects (*n*), Follow Up	Outcome Description	Mean Clinical Outcomes
GSK933776 (Immunoglobulin G subclass 1 mAb directed against the N-terminal amino acid residues 1 through 7 of amyloid b) GlaxoSmithKline, London, United Kingdom	Rosenfeld PJ, et al. 2018 [74]	NCT01342926 Phase 2. Multicenter, randomized, parallel-group, double-masked, placebo-controlled.	Intravenous infusion 3, 6, or 15 mg/kg monthly for 18 months.	191 patients Mean age: 77 years Female: 57% No separate follow-up period reported.	Main outcome: Enlargement in the area of GA measured from colour fundus photographs with reference to fundus autofluorescence images. BCVA and LLBCVA (ETDRS letters).	Intravenous GSK933776 did not slow GA progression or improve visual function, and no effect was seen in relation to CFI genotype.

GA: geographic atrophy, BCVA: best corrected visual acuity, LLBCVA: low-luminance best corrected visual acuity, ETDRS: Early Treatment of Diabetic Retinopathy Study, CFI: complement factor I.

## Data Availability

All primary data and Supplementary Materials supporting the findings of this study are included within the article and its online Supplementary files. Further inquiries may be addressed to the corresponding author. The study protocol was pre-registered on the Open Science Framework (OSF); the registration is currently under embargo and will become publicly accessible upon publication of this manuscript.

## Supplementary Materials

The following supporting information can be downloaded at: https://www.mdpi.com/article/10.3390/pharmaceutics18101269/s1, Table S1. Search strategy through electronic databases, Table S2. Specific criteria applied according to NOS scale, Table S3. ClinPK checklist for quality assessment of the manuscript involving pharmacokinetic studies, Table S4. Analysis of risk of bias using the Newcastle–Ottawa Scale (NOS) for the studies included in the review, Table S5. Characteristics of ClinicalTrials.gov registry entries for geographic atrophy with available data but no associated publications, and PRISMA 2020 Checklist.
