# Supplementary material for: A Systematic Review of Complement-Targeting Therapies for Geographic Atrophy: Insights into Advanced Drug Delivery Systems and Gene-Based Approaches
Source: Pharmaceutics. 2026 Oct 7;18(10):1269. doi: 10.3390/pharmaceutics18101269 (PMC13708414; doi:10.3390/pharmaceutics18101269)
Supplement: Supplementary file 1 [file pharmaceutics-18-01269-s001.zip › pharmaceutics-4500516-supplementary.pdf]

SUPPLEMENTARY MATERIAL

Table S1. Search strategy through electronic databases.

Database	Search strategy
**PubMed**	(("geographic atrophy"[MeSH Terms] OR (("atrophie"[All Fields] OR "atrophy"[MeSH Terms] OR "atrophy"[All Fields] OR "atrophied"[All Fields] OR "atrophies"[All Fields] OR "atrophying"[All Fields]) AND "geographic"[Title/Abstract])) AND ("geographic atrophy"[MeSH Terms] OR "dry macular degeneration"[Title/Abstract]) AND ("complement system proteins"[MeSH Terms] OR "complement"[Title/Abstract])) AND ((adaptiveclinicaltrial[Filter] OR clinicalstudy[Filter] OR clinicaltrial[Filter] OR clinicaltrialphasei[Filter] OR clinicaltrialphaseii[Filter] OR clinicaltrialphaseiii[Filter] OR clinicaltrialphaseiv[Filter] OR comparativestudy[Filter] OR controlledclinicaltrial[Filter] OR equivalencetrial[Filter] OR evaluationstudy[Filter] OR multicenterstudy[Filter] OR observationalstudy[Filter] OR pragmaticclinicaltrial[Filter] OR randomizedcontrolledtrial[Filter]) AND (english[Filter]))
**Embase**	1 geographic atrophy/ (4192)<br>2 geographic atrophy.ti,ab. (3989)<br>3 1 or 2 (5018)<br>4 dry macular degeneration/ (4192)<br>5 dry macular degeneration.ti,ab. (70)<br>6 4 or 5 (4214)<br>7 complement/ (23161)<br>8 complement.ti,ab. (217179)<br>9 7 or 8 (223982)<br>10 3 or 6 (5038)<br>11 9 and 10 (520)<br>12 limit 11 to (humans and english languaje (clinical trial or randomized controlled trial or controlled clinical trial or multicenter study of phase 1 clinical trial or phase 2 clinical trial or phase 3 clinical trial or phase 4 clinical trial)) (107)
**Cochrane**	#1 MeSH descriptor: [Geographic Atrophy] explode all trees 232<br>#2 (geographic atrophy):ti,ab,kw 605<br>#3 (dry macular degeneration):ti,ab,kw 312<br>#4 #1 OR #2 605<br>#5 #4 OR #3 824<br>#6 MeSH descriptor: [Complement System Proteins] explode all trees 909<br>#7 (complement):ti,ab,kw 7289<br>#8 #6 OR #7 7294<br>#9 #8 AND #5 73

Table S2. Specific criteria applied according to Newcastle-Ottawa Scale (NOS) for the studies included in the review.

Selection	1. Representativeness of the exposed cohort.<br>* if<br>a) truly representative of the average in the community.<br>b) somewhat representative of the average in the community.
	2. Selection of the no-exposed cohort.<br>* if drawn from the same community as the exposed cohort.
	3. Ascertainment of exposure.<br>* if adequate selection of cases.
	4. Outcome of interest not present at start of study.<br>* if no efficacy before treatment due to any other treatment.
Comparability	1. Comparability of age of cohorts.<br>* if considers the age the patients.
	2. Comparability of the group size.<br>* if balance in the sample size of the groups.
Outcome	1. Assessment of outcome.<br>*if independent blind assessment and adequately documented.
	2. Follow-up long enough.<br>*if was follow-up long enough for outcomes to occur.
	3. Follow-up complete.<br>*if adequacy of follow up of cohorts (appropriate statistical analysis, all subjects accounted for, description of lost).

Table S3. ClinPK checklist used as a quality assessment tool of the manuscript involving pharmacokinetic studies according to Kanji S et al[1]. Individual scores obtained for each of the three studies evaluated with this tool are also presented.

		Crass RL 2024[2]	Crass RL 2025[3]	Le KN 2015[4]
	**Title/Abstract**			
1	The title identifies the drug(s) and patient population(s) studied	yes	yes	yes
2	The abstract minimally includes the name of the drug(s) studied, route of administration, population in whom it was studied, and results of the primary objective and major clinical pharmacokinetic findings	yes	yes	yes
	**Background**			
3	Pharmacokinetic data (i.e. absorption, distribution, metabolism, excretion) that are known and relevant to the drugs being studied are described	yes	no	no
4	An explanation of the study rationale is provided	yes	yes	yes
5	Specific objectives or hypotheses are provided	yes	yes	yes
	**Methods**			
6	Eligibility criteria of study participants are described	no	no	no
7	Co-administration (or lack thereof) of study drug(s) with other potentially interacting drugs or food within this study is described	no	no	no
8	Drug preparation and administration characteristics, including dose, route, formulation, infusion duration (if applicable), and frequency are described	yes	yes	yes
9	Body fluid or tissue sampling (timing, frequency, and storage) for quantitative drug measurement are described	yes	yes	yes
10	Validation of quantitative bioanalytical methods used in the study are referenced or described if applicable	no	no	no
11	Pharmacokinetic modeling methods and software used are described, including assumptions made regarding the number of compartments and order of kinetics (zero, first, or mixed order)	yes	yes	yes
12	For population pharmacokinetic studies, covariates incorporated into pharmacokinetic models are identified and described	yes	yes	yes
13	Formulas for calculated variables (such as creatinine clearance, body surface area, AUC, and adjusted body weight) are provided or referenced	NA	NA	NA
14	The specific body weight used in drug dosing and pharmacokinetic calculations are reported (i.e., ideal body weight vs. actual body weight vs. adjusted body weight)	NA	NA	NA
15	Statistical methods including software used are described	yes	yes	yes
	**Results**			
16	Study withdrawals or subjects lost to follow- up (or lack thereof) are reported.	no	no	no
17	Quantification of missing or excluded data is provided if applicable.	no	yes	no
18	All relevant variables that may explain inter- and intra-patient pharmacokinetic variability (including: age, sex, end-organ function, ethnicity, weight or BMI, health status or severity of illness, and pertinent co-morbidities) are provided with appropriate measures of variance.	yes	yes	yes
19	Results of pharmacokinetic analyses are reported with appropriate measures of precision (such as range or 95% confidence intervals)	yes	yes	yes
20	Studies in patients receiving extracorporeal drug removal (i.e., dialysis) should report the mode of drug removal, type of filters used, duration of therapy and relevant flow rates.	NA	NA	NA
21	In studies of drug bioavailability comparing two formulations of the same drug, F (bioavailability), AUC, Cmax (maximal concentration) and Tmax (time to maximal concentration) should be reported.	NA	NA	NA
	**Discussion/Conclusion**			
22	Study limitations describing potential sources of bias and imprecision where relevant should be described	yes	no	yes
23	The relevance of study findings (applicability, external validity) is described	yes	yes	yes
24	Funding sources and conflicts of interest for the authors are disclosed.	yes	yes	yes
	**Total**	16/20	14/20	14/20
	**Total**	80%	70%	70%

NA: NA: not available.

[1]Kanji S, et al. Reporting Guidelines for Clinical Pharmacokinetic Studies: The ClinPK Statement. Clin Pharmacokinet. 2015,54:783-95. doi: 10.1007/s40262-015-0236-8.

[2] Crass RL, et al. Population Pharmacokinetics of Pegcetacoplan in Patients with Geographic Atrophy or Neovascular Age-related Macular Degeneration. Ophthalmol Sci 2024,5:100657. doi: 10.1016/j.xops.2024.100657.

[3] Crass RL, Prem K, Gaudreault F, Lusk E, Ribeiro R, Chapel S, Baumal CR. Pharmacokinet-ic/pharmacodynamic analysis of geographic atrophy lesion area in patients receiving pegcetacoplan treat-ment or sham. CPT Pharmacometrics Syst Pharmacol 2025,14,257-267. doi: 10.1002/psp4.13264.

[4] Le KN, et al. Population Pharmacokinetics and Pharmacodynamics of Lampalizumab Administered Intravitreally to Patients with Geographic Atrophy. CPT Pharmacometrics Syst Pharmacol 2015,4,595-604. doi: 10.1002/psp4.12031.

Table S4. Analysis of risk of bias using the Newcastle-Ottawa Scale (NOS) for the studies included in the review.

		SELECTION				COMPARABILITY		OUTCOME			
**No.**	**Study**	**1. Representativeness of the exposed cohort**	**2. Selection of the non-exposed cohort**	**3. Ascertainment of exposure**	**4. Outcome of interest not present at start of study**	**1. Comparability of age of cohorts**	**2. Comparability of group size**	**1. Assessment of outcome**	**2. Follow-up long enough**	**3. Follow-up complete**	**Total star rating (up to 9)**
1	Yehoshua et al., 2014	1	1	1	1	1	1	1	1	0	8
2	Do DB et al et al., 2014	1	1	1	1	1	1	0	1	0	**7**
3	Yaspan BL et al., 2017	1	1	1	1	1	1	1	1	1	**9**
4	Holz FG et al., 2018	1	1	1	1	1	1	1	1	1	**9**
5	Liao DS et al., 2020	1	1	1	1	1	1	1	1	0	**8**
6	Jaffe GJ et al., 2021	1	1	1	1	1	0	1	1	1	**8**
7	Wykoff CC et al., 2021	0	0	1	1	0	1	1	1	1	**6**
8	Steinle NC et al., 2021	0	0	1	1	1	1	1	1	1	**7**
9	Wykoff CC et al., 2022	1	0	1	1	1	0	0	0	1	**5**
10	Nittala MN et al., 2022	0	0	1	1	0	0	1	1	1	**5**
11	Pfau M et al., 2022	0	0	1	1	1	1	0	1	1	**6**
12	Mai J et al., 2022	0	0	1	1	1	1	1	1	0	**6**
13	Riedl S et al., 2022	0	0	1	1	0	1	1	1	1	**6**
14	Khanani AM et al., 2022	1	0	1	1	1	1	1	1	0	**7**
15	Patel SS et al., 2023	1	1	1	1	1	0	1	1	0	**7**
16	Khanani AM et al., 2023	1	1	1	1	1	1	1	1	1	**9**
17	Heier JS et al., 2023	1	1	1	1	1	1	1	1	0	**8**
18	Edmonds R et al., 2023	0	0	1	1	1	1	1	1	1	**7**
19	Danzig CJ et al., 2024	0	0	1	1	1	1	1	1	1	**7**
20	Rosenfeld PJ et al., 2018	1	1	1	1	1	1	1	1	1	**9**
21	Schmidt-Erfurth U et al., 2024	0	0	1	1	1	0	1	1	1	**6**
22	Heier JS et al., 2024	1	0	1	1	1	0	1	1	1	**7**
23	Corradetti G et al., 2025	0	0	1	1	1	0	1	1	0	**5**
24	Goldberg RA et al., 2025	1	1	1	1	1	1	0	1	1	**8**
25	Wykoff CC et al., 2025	1	1	1	1	1	1	0	1	1	**8**
26	Rush RB et al., 2025	1	0	1	1	1	1	0	1	0	**6**
27	McCaleb ML et al., 2025	0	0	1	1	1	1	1	1	0	**6**
28	Khanani AM et al., 2026	1	1	1	1	1	1	1	1	1	**9**

Studies are ordered from oldest to newest by publication year. Studies including post hoc analyses not prespecified in the protocol received no star in the comparability domain, due to the increased risk of data-driven results.

List of references (same order as in the table)

1. Yehoshua Z, de Amorim Garcia Filho CA, Nunes RP, Gregori G, Penha FM, Moshfeghi AA, Zhang K, Sadda S, Feuer W, Rosenfeld PJ. Systemic complement inhibition with eculizumab for geographic atrophy in age-related macular degeneration: the COMPLETE study. *Ophthalmology* **2014**,121,693-701. doi: 10.1016/j.ophtha.2013.09.044.
2. Do DV, Pieramici DJ, van Lookeren Campagne M, Beres T, Friesenhahn M, Zhang Y, Strauss EC; Phase Ia Investigators. A phase ia dose-escalation study of the anti-factor D monoclonal antibody fragment FCFD4514S in patients with geographic atrophy. *Retina* **2014**,34,:313-20. doi: 10.1097/IAE.0b013e3182979ddd.
3. Yaspan BL, Williams DF, Holz FG, Regillo CD, Li Z, Dressen A, van Lookeren Campagne M, Le KN, Graham RR, Beres T, Bhangale TR, Honigberg LA, Smith A, Henry EC, Ho C, Strauss EC; MAHALO Study Investigators. Targeting factor D of the alternative complement pathway reduces geographic atrophy progression secondary to age-related macular degeneration. *Sci Transl Med* **2017**,9,eaaf1443. doi: 10.1126/scitranslmed.aaf1443.
4. Holz FG, Sadda SR, Busbee B, Chew EY, Mitchell P, Tufail A, Brittain C, Ferrara D, Gray S, Honigberg L, Martin J, Tong B, Ehrlich JS, Bressler NM; Chroma and Spectri Study Investigators. Efficacy and Safety of Lampalizumab for Geographic Atrophy Due to Age-Related Macular Degeneration: Chroma and Spectri Phase 3 Randomized Clinical Trials. *JAMA Ophthalmol* **2018**, 136,666-677. doi: 10.1001/jamaophthalmol.2018.1544.
5. Liao DS, Grossi FV, El Mehdi D, Gerber MR, Brown DM, Heier JS, Wykoff CC, Singerman LJ, Abraham P, Grassmann F, Nuernberg P, Weber BHF, Deschatelets P, Kim RY, Chung CY, Ribeiro RM, Hamdani M, Rosenfeld PJ, Boyer DS, Slakter JS, Francois CG. Complement C3 Inhibitor Pegcetacoplan for Geographic Atrophy Secondary to Age-Related Macular Degeneration: A Randomized Phase 2 Trial. *Ophthalmology* **2020**,127,186-195. doi: 10.1016/j.ophtha.2019.07.011.
6. Jaffe GJ, Westby K, Csaky KG, Monés J, Pearlman JA, Patel SS, Joondeph BC, Randolph J, Masonson H, Rezaei KA. C5 Inhibitor Avacincaptad Pegol for Geographic Atrophy Due to Age-Related Macular Degeneration: A Randomized Pivotal Phase 2/3 Trial. *Ophthalmology* **2021**, 128,576-586. doi: 10.1016/j.ophtha.2020.08.027.
7. Wykoff CC, Rosenfeld PJ, Waheed NK, Singh RP, Ronca N, Slakter JS, Staurenghi G, Monés J, Baumal CR, Saroj N, Metlapally R, Ribeiro R. Characterizing New-Onset Exudation in the Randomized Phase 2 FILLY Trial of Complement Inhibitor Pegcetacoplan for Geographic Atrophy. *Ophthalmology* **2021**,128,1325-1336. doi: 10.1016/j.ophtha.2021.02.025.
8. Steinle NC, Pearce I, Monés J, Metlapally R, Saroj N, Hamdani M, Ribeiro R, Rosenfeld PJ, Lad EM. Impact of Baseline Characteristics on Geographic Atrophy Progression in the FILLY Trial Evaluating the Complement C3 Inhibitor Pegcetacoplan. *Am J Ophthalmol* **2021**,227,116-124. doi: 10.1016/j.ajo.2021.02.031.
9. Wykoff CC, Hershberger V, Eichenbaum D, Henry E, Younis HS, Chandra P, Yuan N, Solloway M, DePaoli A. Inhibition of Complement Factor 3 in Geographic Atrophy with NGM621: Phase 1 Dose-Escalation Study Results. *Am J Ophthalmol* **2022**,235,131-142. doi: 10.1016/j.ajo.2021.08.018.
10. Nittala MG, Metlapally R, Ip M, Chakravarthy U, Holz FG, Staurenghi G, Waheed N, Velaga SB, Lindenberg S, Karamat A, Koester J, Ribeiro R, Sadda S. Association of Pegcetacoplan With Progression of Incomplete Retinal Pigment Epithelium and Outer Retinal Atrophy in Age-Related Macular Degeneration: A Post Hoc Analysis of the FILLY Randomized Clinical Trial. *JAMA Ophthalmol* **2022**,140,243-249. doi: 10.1001/jamaophthalmol.2021.6067.
11. Pfau M, Schmitz-Valckenberg S, Ribeiro R, Safaei R, McKeown A, Fleckenstein M, Holz FG. Association of complement C3 inhibitor pegcetacoplan with reduced photoreceptor degeneration beyond areas of geographic atrophy. *Sci Rep* **2022**,12,17870. doi: 10.1038/s41598-022-22404-9.
12. Mai J, Riedl S, Reiter GS, Lachinov D, Vogl WD, Bogunovic H, Schmidt-Erfurth U. Comparison of Fundus Autofluorescence Versus Optical Coherence Tomography-based Evaluation of the Therapeutic Response to Pegcetacoplan in Geographic Atrophy. *Am J Ophthalmol* **2022**,244,175-182. doi: 10.1016/j.ajo.2022.06.023.
13. Riedl S, Vogl WD, Mai J, Reiter GS, Lachinov D, Grechenig C, McKeown A, Scheibler L, Bogunović H, Schmidt-Erfurth U. The Effect of Pegcetacoplan Treatment on Photoreceptor Maintenance in Geographic Atrophy Monitored by Artificial Intelligence-Based OCT Analysis. *Ophthalmol Retina* **2022**,6,1009-1018. doi: 10.1016/j.oret.2022.05.030.

14. Khanani AM, Maturi RK, Bagheri N, Bakall B, Boyer DS, Couvillion SS, Dhoot DS, Holekamp NM, Jamal KN, Marcus DM, Pieramici D, Aziz AA, Patki KC, Bridges WZ Jr, Barone SB. A Phase I, Single Ascending Dose Study of GEM103 (Recombinant Human Complement Factor H) in Patients with Geographic Atrophy. *Ophthalmol Sci* **2022**,2,100154. doi: 10.1016/j.xops.2022.100154.
15. Patel SS, Lally DR, Hsu J, Wykoff CC, Eichenbaum D, Heier JS, Jaffe GJ, Westby K, Desai D, Zhu L, Khanani AM. Avacincaptad pegol for geographic atrophy secondary to age-related macular degeneration: 18-month findings from the GATHER1 trial. *Eye (Lond)* **2023**,37,3551-3557. doi: 10.1038/s41433-023-02497-w. Erratum in:. *Eye (Lond)* **2023**,37,3705. doi: 10.1038/s41433-023-02548-2.
16. Khanani AM, Patel SS, Staurenghi G, Tadayoni R, Danzig CJ, Eichenbaum DA, Hsu J, Wykoff CC, Heier JS, Lally DR, Monés J, Nielsen JS, Sheth VS, Kaiser PK, Clark J, Zhu L, Patel H, Tang J, Desai D, Jaffe GJ; GATHER2 trial investigators. Efficacy and safety of avacincaptad pegol in patients with geographic atrophy (GATHER2): 12-month results from a randomised, double-masked, phase 3 trial. *Lancet* **2023**,402,1449-1458. doi: 10.1016/S0140-6736(23)01583-0.
17. Heier JS, Lad EM, Holz FG, Rosenfeld PJ, Guymer RH, Boyer D, Grossi F, Baumal CR, Korobelnik JF, Slakter JS, Waheed NK, Metlapally R, Pearce I, Steinle N, Francone AA, Hu A, Lally DR, Deschatelets P, Francois C, Bliss C, Staurenghi G, Monés J, Singh RP, Ribeiro R, Wykoff CC; OAKS and DERBY study investigators. Pegcetacoplan for the treatment of geographic atrophy secondary to age-related macular degeneration (OAKS and DERBY): two multicentre, randomised, double-masked, sham-controlled, phase 3 trials. *Lancet* **2023**,402,1434-1448. doi: 10.1016/S0140-6736(23)01520-9.
18. Edmonds R, Steffen V, Honigberg LA, Chang MC. Alternative Complement Pathway Inhibition by Lampalizumab: Analysis of Data From Chroma and Spectri Phase III Clinical Trials. *Ophthalmol Sci* **2023**,3,100286. doi: 10.1016/j.xops.2023.100286.
19. Danzig CJ, Khanani AM, Kaiser PK, Chang MA, Kovach JL, Lally DR, Rachitskaya A, Sheth VS, Vajzovic L, Clark J, Tang J, Zhu L, Desai D, Chakravarthy U. Vision Loss Reduction with Avacincaptad Pegol for Geographic Atrophy: A 12-Month Post Hoc Analysis of the GATHER1 and GATHER2 Trials. *Ophthalmol Retina* **2024**,8,1052-1060. doi: 10.1016/j.oret.2024.04.023.
20. Rosenfeld PJ, Berger B, Reichel E, Danis RP, Gress A, Ye L, Magee M, Parham LR, McLaughlin MM. A Randomized Phase 2 Study of an Anti-Amyloid β Monoclonal Antibody in Geographic Atrophy Secondary to Age-Related Macular Degeneration. *Ophthalmol Retina* **2018**,2,1028-1040. doi: 10.1016/j.oret.2018.03.001.
21. Schmidt-Erfurth U, Mai J, Reiter GS, Riedl S, Vogl WD, Sadeghipour A, McKeown A, Foos E, Scheibler L, Bogunovic H. Disease Activity and Therapeutic Response to Pegcetacoplan for Geographic Atrophy Identified by Deep Learning-Based Analysis of OCT. *Ophthalmology* **2025,**132,181-193. doi: 10.1016/j.ophtha.2024.08.017.
22. Heier JS, Cohen MN, Chao DL, Pepio A, Shiraga Y, Capuano G, Rogers A, Ackert J, Sen HN, Csaky K. Phase 1 Study of JNJ-81201887 Gene Therapy in Geographic Atrophy Secondary to Age-Related Macular Degeneration. *Ophthalmology* **2024,**131,1377-1388. doi: 10.1016/j.ophtha.2024.06.013.
23. Corradetti G, Karamat A, Srinivas S, Lindenberg S, Velaga SB, Corvi F, Attiku Y, Nittala MG, Desai D, Zhu L, Abulon D, Sadda SR. Progression to complete retinal pigment epithelium and outer retinal atrophy (cRORA): post hoc analysis of the GATHER1 trial. *Graefes Arch Clin Exp Ophthalmol* **2025**,263,669-677. doi: 10.1007/s00417-024-06676-7.
24. Goldberg RA, Boyer DS, Holz FG, MacCumber MW, Garg SJ, Brown DM, Lad EM, Steinle N, Rishi PS, Pearce I, Ach T, Ribeiro R, Li C, Jones D, Tsuboi M, Ferrone PJ, Baumal CR, Wykoff CC. Pegcetacoplan for Geographic Atrophy Over 30 Months: Data From OAKS, DERBY, and the GALE Long-Term Extension Study. *Ophthalmic Surg Lasers Imaging Retina* **2025**,56,398-406. doi: 10.3928/23258160-20250217-01.
25. Wykoff CC, Holz FG, Chiang A, Boyer D, Dhoot DS, Loewenstein A, Mones J, Heier J, Abbey AM, Singerman LJ, Vajzovic L, Lin J, Li C, Vilupuru A, Baumal CR; OAKS, DERBY, and GALE Investigators. Pegcetacoplan Treatment for Geographic Atrophy in Age-Related Macular Degeneration Over 36 Months: Data From OAKS, DERBY, and GALE. *Am J Ophthalmol* **2025**,276,350-364. doi: 10.1016/j.ajo.2025.04.016.

26. Rush RB, Klein W, Reinauer RM. Real-World Outcomes with Complement Inhibitors for Geographic Atrophy: A Comparative Study of Pegacetacoplan versus Avacincaptad Pegol. *Clin Ophthalmol* **2025,**19,1167-1174. doi: 10.2147/OPTH.S518398.
27. McCaleb ML, Hughes SG, Grossman TR, Frazer-Abel A, Jung B, Yin L, Henry SP, Monia BP, Schneider E, Geary R, Brice GT. Inhibiting the alternative pathway of complement by reducing systemic complement factor B: Randomized, double-blind, placebo-controlled phase 1 studies with Sefaxersen. *Immunobiology* **2025**,230,152876. doi: 10.1016/j.imbio.2025.152876.
28. Khanani AM, Danzig CJ, Heier JS, Jaffe GJ, Kaiser PK, Lally DR, Patel SS, Vajzovic L, Weng CY, Patel H, Clark J, Desai D, Luo D, Henry E, Holz FG; GATHER2 Trial Investigators. Avacincaptad Pegol for Geographic Atrophy Secondary to Age-Related Macular Degeneration: Two-Year Efficacy and Safety Results from the GATHER2 Phase 3 Trial. *Ophthalmology* **2026**,133,:451-465. doi: 10.1016/j.ophtha.2025.12.011.

Table S5. Characteristics of ClinicalTrials.gov registry entries for geographic atrophy with available data but no associated publications.

NCT Number	Title	Date	Drug	Results	Completed	Route of administration and dose	Phase
NCT04465955	A Phase 2 Multicenter, Randomized, Double-Masked, Sham-Controlled Study of the Safety and Efficacy of Intravitreal Injections of NGM621 in Subjects with Geographic Atrophy (GA) Secondary to Age-Related Macular Degeneration (AMD)	10/07/2020-01/07/2025	NGM621 is a humanized immunoglobulin G1 monoclonal antibody, it binds to C3 and inhibits its cleavage to C3a and C3b<br><br>vs sham	Yes	Yes	Intravitreal injection<br><br>Dose 1 every 4 weeks. Dose 2 every 8 weeks	3
NCT01603043	A Multicenter, Randomized, Single-Masked, Sham-Controlled, Proof-of-Concept Study of Intravitreal AL-78898A in Patients with Bilateral Geographic Atrophy (GA) Associated with Age-Related Macular Degeneration (AMD)	06/2012-01/2014	AL-78898A binds to C3, preventing its cleavage into active fragments, thereby reducing complement activation and subsequent ocular inflammation	No	Terminated	Intravitreal injection<br><br>0.4 mg per 50 µL per month for up to 12 months	2
NCT05019521	A Study of Danicopan in Participants with Geographic Atrophy Secondary to Age-Related Macular Degeneration	23/08/2021-05/09/2025	Danicopan (ALXN2040) is an inhibitor of complement Factor D<br><br>vs sham	Yes	Terminated	Oral tablet<br><br>100 mg twice daily (bid), 200 mg bid, 400 mg once daily up to 52 weeks	2
NCT02288559	A Phase II, Multicenter, Randomized, Single-Masked, Sham Injection-Controlled Exposure-Response Study of Lampalizumab Intravitreal Injections Administered Every Two Weeks or Every Four Weeks to Patients with Geographic Atrophy	30/03/2015-25/09/2019	Lampalizumab specifically inhibits complement factor D<br><br>vs sham	Yes	Yes	Intravitreal injection<br><br>10 milligrams lampalizumab every two weeks during the safety run-in period. Participants receive 10 mg dose every two weeks during the 24-week treatment period. Participants receive 10 mg dose every four weeks during the 24-week treatment period	2

NCT Number	Title	Date	Drug	Results	Completed	Route of administration and dose	Phase
NCT01602120	A Multicenter, Open-Label Extension Study to Evaluate the Long-Term Safety and Tolerability of Lampalizumab (FCFD4514S) in Patients with Geographic Atrophy Who Have Completed Genentech-Sponsored Lampalizumab Studies	29/05/2012-11/09/2019	Lampalizumab specifically inhibits complement factor D<br><br>vs sham	Terminated	Yes	Intravitreal injection<br><br>Initially every 4 weeks or every 8 weeks (per parent study), then every 4 weeks for the remainder of the extension study	2
NCT02745119	A Multicenter, Open-Label Extension Study to Evaluate the Long-Term Safety and Tolerability of Lampalizumab in Patients with Geographic Atrophy Secondary to Age-Related Macular Degeneration Who Have Completed a Roche-Sponsored Study	21/07/2016-03/06/2019	Lampalizumab specifically inhibits complement factor D	Terminated	Yes	Intravitreal injection<br><br>10 mg every 4 or 6 weeks up to 48 weeks	3
NCT06541704	A Multicenter, Randomized, Double-Masked, Placebo-Controlled Phase 3 Study of the Efficacy, Safety, and Tolerability of Subcutaneously Administered Pozelimab in Combination with Cemdisiran or Cemdisiran Alone in Participants with Geographic Atrophy Secondary to Age-Related Macular Degeneration	30/10/2024 to present	Pozelimab, a monoclonal antibody, inhibit complement component C5. Cemdisiran is a RNA interference (RNAi) therapeutic designed to silence the complement C5 gene	Recruiting	No	Subcutaneous injection<br><br>Pozelimab + Cemdisiran treatment group or Cemdisiran monotherapy every 4 weeks during 104 weeks	3
NCT05536297	An Open-label Extension (OLE) Phase 3 Trial to Assess the Safety of Intravitreal Administration of Avacincaptad Pegol (Complement C5 Inhibitor) in Patients with Geographic Atrophy Who Previously Completed Phase 3 Study ISEE2008 (GATHER2)	26/09/2022-18/05/2026	Avacincaptad Pegol, complement C5 inhibitor	Yes	Yes	Intravitreal injection<br><br>2 mg monthly up to month 17	3
NCT01255462	A Multicenter, Open Label, Single Ascending Dose Study to Assess the Safety, Tolerability, and Serum Pharmacokinetics of Intravitreal LFG316 in Patients with Advanced Age-related Macular Degeneration	11/2010-05/2012	LFG316 (Tesidolumab, monoclonal antibody anti-C5)	Completed	No	Intravitreal injection<br><br>0.15, 0.5, 1.5 or 5 mg (single dose)	1

NCT Number	Title	Date	Drug	Results	Completed	Route of administration and dose	Phase
NCT01527500	A Multicenter, Randomized, Sham-control, Proof-of-concept Study of Intravitreal LFG316 in Patients with Geographic Atrophy Associated with Age-related Macular Degeneration	25/01/2012-05/01/2021	LFG316 (Tesidolumab, monoclonal antibody anti-C5)<br><br>vs sham	Completed	Yes	Intravitreal injection<br><br>5 mg/50 µL solution or 10 mg/100 µL injection monthly up to 18 months	2
NCT03815825	GOLDEN STUDY: A Study to Assess Safety and Efficacy of Multiple Doses of IONIS-FB-LRx in Participants with Geographic Atrophy Secondary to Age-Related Macular Degeneration (AMD)	04/03/2019-07/03/2025	IONIS-FB-LRx, (antisense Inhibitor of Complement Factor B)<br><br>vs sham	No	Yes	Subcutaneous injection<br><br>In the first stage, participants received one of three dose levels administered SC every 4 weeks until Week 45. In the second stage, participants were randomized to one of two expanded cohorts and continued receiving SC administration every 4 weeks until Week 45.	2
NCT03446144	A Phase 2, Placebo-Controlled, Double-Masked Study to Assess Safety and Efficacy of ISIS 696844, an Antisense Inhibitor of Complement Factor B, in Patients with Geographic Atrophy Secondary (GA) to Age-Related Macular Degeneration (AMD)	16/03/2018-13/08/2019	IONIS-FB-Lrx (antisense inhibitor of complement factor B)<br><br>vs sham procedure (sterile saline 0.9%)	No	Withdrawn	Subcutaneous injection<br><br>Single dose once weekly for weeks 1-3, and every other week or less frequently until week 70.	2
NCT05230537	A Randomized, Participant and Investigator Masked, Placebo-controlled, Multicenter, Proof-of-concept Study to Assess the Safety and Efficacy of LNP023 (Iptacopan) in Patients with Early and Intermediate Age-related Macular Degeneration	17/02/2022-03/07/2025	Iptacopan: factor B inhibitor<br><br>vs sham	Active, not recruiting	No	Oral capsules	2
NCT03846193	FOCUS: An Open Label First in Human Phase I/II Multicentre Study to Evaluate the Safety, Dose Response and Efficacy of	17/12/2018 to present	GT005/ Device: Orbit™ Subretinal Delivery System	Terminated	Yes	GT005 was administered as a single subretinal dose. In Parts 1 and 2,	1 and 2

NCT Number	Title	Date	Drug	Results	Completed	Route of administration and dose	Phase
	GT005 Administered as a Single Subretinal Injection in Subjects With Macular Atrophy Due to AMD		A recombinant non-replicating Adeno-Associated Viral (AAV) vector encoding a human complement factor I			delivery was performed via a transvitreal procedure, whereas in Parts 3 and 4, GT005 was delivered via suprachoroidal cannulation using the Orbit Subretinal Delivery System (SDS). Three dose levels were evaluated: low dose (2×10^{10} vector genomes [vg]), medium dose (5×10^{10} vg), and high dose (2×10^{11} vg)	
NCT05481827	ORACLE: A Long-term Follow-up Study to Evaluate the Safety of GT005 in Participants with Geographic Atrophy Secondary to Age-related Macular Degeneration Treated in a Gyroscope-sponsored Antecedent Study	12/07/2022 to present	GT005: recombinant adeno-associated viral serotype 2 (AAV2) vector encoding for human complement factor I.	No	Enrolling by invitation	No dose administered.<br><br>Follow up study of participants previously treated with GT005	2
NCT04437368	EXPLORE: A Phase II, Outcomes Assessor-masked, Multicentre, Randomised Study to Evaluate the Safety and Efficacy of Two Doses of GT005 Administered as a Single Subretinal Injection in Subjects with Geographic Atrophy Secondary to Age-related Macular Degeneration	14/07/2020-05/04/2024	A recombinant non-replicating Adeno-Associated Viral (AAV) vector encoding a human complement factor I.	Terminated	Yes	Subretinal injection<br><br>Single dose via suprachoroidal cannulation using the Orbit Subretinal Delivery System (SDS). Two dose levels were evaluated: low dose (2×10^{10} vg) and high dose (2×10^{11} vg)	2
NCT04566445	HORIZON: A Phase II, Open-label, Outcomes-assessor Masked, Multicentre,	25/09/2020-27/01/2026	A recombinant non-replicating Adeno-	Terminated	Yes	Subretinal injection	2

NCT Number	Title	Date	Drug	Results	Completed	Route of administration and dose	Phase
	Randomised, Controlled Study to Evaluate the Safety and Efficacy of Two Doses of GT005 Administered as a Single Subretinal Injection in Subjects with Geographic Atrophy Secondary to Dry Age-related Macular Degeneration		Associated Viral (AAV) vector encoding a human complement factor I.			Two dose levels were evaluated: medium dose (5×10^{10} vector genomes [vg]) and high dose (2×10^{11} vg)	
NCT04656561	A Phase 2, Multicenter, Randomized, Parallel-Group, Double-Masked, 4-Arm, Sham-Controlled Study of the Efficacy, Safety, and Tolerability of ANX007 Administered by Intravitreal Injection in Patients With Geographic Atrophy (GA) Secondary to Age-Related Macular Degeneration (AMD) - The ARCHER Study	20/08/2020-21/08/2024	ANX007: inhibitor of complement C1q, the initiating molecule of the classical complement cascade. It is involved in synapse elimination and neuronal loss in neurodegenerative diseases<br><br>vs sham	No	Yes	Intravitreal injection<br><br>Every month or every other month up to 12 months	2
NCT06510816	A Phase 3, Multicenter, Randomized, Parallel-Group, Double-Masked, 2-Arm, Sham Controlled Study of the Efficacy, Safety, and Tolerability of ANX007 (Vonaprument) Administered by Intravitreal Injection in Participants With Dry Age-Related Macular Degeneration With Geographic Atrophy	03/07/2024-04/05/2026	ANX007: inhibitor of complement C1q, the initiating molecule of the classical complement cascade. It is involved in synapse elimination and neuronal loss in neurodegenerative diseases<br><br>vs sham	No	Active, not recruiting	Intravitreal injection<br><br>Every month during the 24-month treatment period	3
NCT05811351	A Phase 2b, Randomized, Double-masked, Multicenter, Dose-ranging, Sham-controlled Clinical Trial to Evaluate Intravitreal JNJ-81201887 (AAVCAGsCD59) Compared to Sham Procedure for the Treatment of Geographic Atrophy (GA) Secondary to Age-related Macular Degeneration (AMD)	07/03/2023-13/03/2026	JNJ-81201887 delivers a gene into the retina using an AAV2 viral vector, enabling the production of sCD59, a protein that blocks MAC formation.<br><br>vs sham	No	Completed	Intravitreal injection.<br><br>Low and high dose levels. Corticosteroid prophylaxis with oral prednisone and intravitreal triamcinolone was used as part of the treatment regimen	2

NCT Number	Title	Date	Drug	Results	Completed	Route of administration and dose	Phase
NCT06635148	Long-term Extension Study for Participants with Geographic Atrophy (GA) Secondary to Age-related Macular Degeneration (AMD) in JNJ-81201887 Parent Clinical Studies	19/09/2024 to present	JNJ-81201887 delivers a gene into the retina using an AAV2 viral vector, enabling the production of sCD59, a protein that blocks MAC formation<br><br>vs sham	Recruiting	No	No study intervention will be administered as part of this study, follow-up of participants previously treated with JNJ-81201887 by intravitreal injection in parent studies	2
NCT04358471	A Phase 2 Multi-Center Trial Evaluating Intravitreal AAVCAGsCD59 Compared to Sham Injection for the Treatment of Advanced Dry Age-Related Macular Degeneration (AMD) With Geographic Atrophy (GA)	31/07/2021-10/05/2021	AAVCAGsCD59, adeno-associated virus (AAV)-based gene therapy that delivers a soluble form of CD59, an endogenous inhibitor of the terminal complement pathway that prevents assembly of the membrane attack complex<br><br>vs sham	No	Withdrawn	Intravitreal injection AAVACGsCD59 at a dose of $1.071x10^{12}$ vg or $3.56x10^{11}$ vg administered once on Day 0	2
NCT01835015	A Multi-Center, Open-Label, Single Ascending Dose Study to Assess the Safety, Tolerability, and Serum Pharmacokinetics of Intravitreal CLG561 in Subjects with Advanced Age-Related Macular Degeneration	05/2013-03/2016	CLG561 is a human antibody fragment (Fab) that targets and neutralizes properdin, a positive regulator of the alternative pathway of the complement system	Yes	Yes	Intravitreal injection<br><br>Injection on day 1 across five escalating concentration levels (A-E). Injection volumes were 50 µL for dose levels A-D and 100 µL for dose level E	1
NCT02515942	A Randomized, Multi-Center, Single Masked, Sham Controlled, Proof-of-Concept Study of Intravitreal CLG561 as a Monotherapy and in Combination with LFG316 in Subjects with Geographic Atrophy	25/09/2015-30/05/2019	CLG561 is a human antibody fragment (Fab) that targets and neutralizes properdin, a positive regulator of the alternative pathway of the complement system.	Yes	Yes	Intravitreal injection<br><br>10 mg CLG561 or 5 mg CLG561+ 5 mg LFG316 every 28 days for a total of 12 injections	2

NCT Number	Title	Date	Drug	Results	Completed	Route of administration and dose	Phase
			LFG316 (a C5 complement inhibitor)<br><br>Vs sham				
NCT04615325	A Phase 1a, Multicenter, Open-label, Single-dose, Dose-escalation Study of the Safety, Tolerability, Pharmacokinetics, and Immunogenicity of Intravitreal Injections of RO7303359 in Patients with Geographic Atrophy Secondary to Age-related Macular Degeneration	08/12/2020-01/03/2023	RO7303359 is a monoclonal antibody designed to inhibit properdin, a protein that positively regulates the alternative pathway of the complement system	No	Yes	Intravitreal injection<br><br>Single ascending dose stage with escalating dose cohorts, followed by expansion cohorts receiving the maximum tolerated dose or maximum tested dose	1a
NCT05380492	Phase 1/2a Dose Escalation Study of VOY-101 in Patients with Advanced Non-Neovascular Age-Related Macular Degeneration	17/11/2022-11/2025	VOY-101, VOY-101 is an adeno-associated virus (AAV)-based gene therapy designed for the treatment of dry age-related macular degeneration (AMD). Its mechanism of action relies on the supplementation of FHL-1, a variant of complement factor H, which plays a crucial role in regulating the complement system	No	Unknown status	Intravitreal injection<br><br>Low, mid and high single dose	1, 2 a
NCT06765980	A Phase 1/2, First-in-Human, Multi-Arm, Dose-Escalation Study to Evaluate the Safety, Tolerability, and Efficacy of an Adeno-associated Virus Vector VV-14295 Administered Suprachoroidally with the Everads Injector in AdultS with GeographIc Atrophy Secondary to Age-related Macular DegeneratiON (the VISION Study)	03/2025 to present	VV-14295 delivers a complement-regulatory transgene via an AAV vector, aiming to inhibit excessive complement activation and reduce downstream membrane attack complex (MAC)-mediated retinal cell injury	Recruiting	No	Suprachoroidal injection<br><br>Low or high dose.	1, 2

NCT Number	Title	Date	Drug	Results	Completed	Route of administration and dose	Phase
NCT05170048	A Phase 2, Parallel, Randomized, Open-label, Controlled Study of EG-301 150 mg Daily in Patients With Nonfocal Geographic Atrophy Secondary to Dry-AMD	06/2025 to present	EG-301: inhibitor of the retinal pigment epithelium complement activation	Not yet recruiting	No	Oral tablet<br><br>150 mg once daily up to 24 months	2
NCT05839041	A Single and Multiple Dose Study to Evaluate Safety, Pharmacokinetics, and Treatment Effect of Intravitreal AVD-104 in Participants with Geographic Atrophy Secondary to Age-related Macular Degeneration	02/05/2023 to present	AVD-104 (recombinant monoclonal antibody fragment designed to inhibit the classical complement pathway by targeting C1q, the initiating component of the classical pathway)<br><br>vs avacincaptad pegol	Active, not recruiting	No	Intravitreal injection<br><br>Single escalating doses in Part 1, followed by high-dose bimonthly or low-dose monthly injections for 24 months in Part 2	2
NCT04246866	A Phase 1, Multicenter, Open-label, Single-dose, Dose-escalation Study in Patients With Geographic Atrophy (GA) Secondary to Dry Age-related Macular Degeneration (AMD) to Evaluate the Safety, Tolerability, Pharmacodynamics, and Immunogenicity of Intravitreal Injections of GEM103	19/12/2019-11/02/2021	GEM103: recombinant Human Complement Factor H	No	Yes	Intravitreal injection<br><br>Single dose of 4 dose cohorts	1
NCT04643886	A Multicenter, Open-label, Multiple Dose Study in Patients with Geographic Atrophy Secondary to Dry Age-related Macular Degeneration to Evaluate the Safety, Tolerability, Pharmacodynamics, and Immunogenicity of Repeat Intravitreal Injections of GEM103	30/07/2020-23/09/2022	GEM103: recombinant Human Complement Factor H	No	Terminated	Intravitreal injection<br><br>Multiple-dose treatment	2
